# Regulatory T cells alleviate myelin loss and cognitive dysfunction by regulating neuroinflammation and microglial pyroptosis via TLR4/MyD88/NF-κB pathway in LPC-induced demyelination

**DOI:** 10.1186/s12974-023-02721-0

**Published:** 2023-02-18

**Authors:** Yao Wang, Dilinuer Sadike, Bo Huang, Ping Li, Qiao Wu, Na Jiang, Yongkang Fang, Guini Song, Li Xu, Wei Wang, Minjie Xie

**Affiliations:** 1 Department of Neurology, Tongji Hospital, Tongji Medical College, Huazhong University of Science and Technology, No. 1095 Jiefang Avenue, Wuhan, 430030 People’s Republic of China; 2 Department of Radiology, Tongji Hospital, Tongji Medical College, Huazhong University of Science and Technology, Wuhan, 430030 People’s Republic of China

**Keywords:** Tregs, Pyroptosis, Demyelination, Microglia, Neuroinflammation, TLR4/MyD88

## Abstract

Demyelination occurs in multiple central nervous system (CNS) disorders and is tightly associated with neuroinflammation. Pyroptosis is a form of pro-inflammatory and lytic cell death which has been observed in CNS diseases recently. Regulatory T cells (Tregs) have exhibited immunoregulatory and protective effects in CNS diseases. However, the roles of Tregs in pyroptosis and their involvement in LPC-induced demyelination have not been explicated. In our study, Foxp3-diphtheria toxin receptor (DTR) mice treated with diphtheria toxin (DT) or PBS were subjected to two-site lysophosphatidylcholine (LPC) injection. Immunofluorescence, western blot, Luxol fast blue (LFB) staining, quantitative real-time PCR (qRT-PCR) and neurobehavior assessments were performed to evaluate the severity of demyelination, neuroinflammation and pyroptosis. Pyroptosis inhibitor was further used to investigate the role of pyroptosis in LPC-induced demyelination. RNA-sequencing was applied to explore the potential regulatory mechanism underlying the involvement of Tregs in LPC-induced demyelination and pyroptosis. Our results showed that depletion of Tregs aggravated microgliosis, inflammatory responses, immune cells infiltration and led to exacerbated myelin injury as well as cognitive defects in LPC-induced demyelination. Microglial pyroptosis was observed after LPC-induced demyelination, which was aggravated by Tregs depletion. Inhibition of pyroptosis by VX765 reversed myelin injury and cognitive function exacerbated by Tregs depletion. RNA-sequencing showed TLR4/myeloid differentiation marker 88 (MyD88) as the central molecules in Tregs-pyroptosis pathway, and refraining TLR4/MyD88/NF-κB pathway alleviated the aggravated pyroptosis induced by Tregs depletion. In conclusion, our findings for the first time indicate that Tregs alleviate myelin loss and improve cognitive function by inhibiting pyroptosis in microglia via TLR4/MyD88/NF-κB pathway in LPC-induced demyelination.

**Supplementary Information:**

The online version contains supplementary material available at 10.1186/s12974-023-02721-0.

## Background

In the CNS, the integrity of myelin is vital for saltatory conduction of electron signals and maintaining normal neuronal functions [1]. Loss of myelin occurs in numerous CNS diseases including autoimmune diseases, cerebrovascular diseases, neurodegenerative diseases and injuries of brain and spinal cord. Demyelination can impair axonal conduction and block nutritional support for axons, finally leading to functional deficits [2]. Among large amounts of studies which have focused on the etiology and pathophysiology of demyelination, neuroinflammation has been shown to be crucial [3]. Neuroinflammation is a kind of sterile inflammation and has been acknowledged to aggravate demyelination and inhibit remyelination in demyelinated diseases [2, 4]. Pro-inflammatory cytokines from microglia, astrocytes and infiltrating immune cells such as interleukin-1β (IL-1β), tumor necrosis factor-α (TNF-α) and interferon-γ (IFN-γ) have been proved to induce demyelination [5]. Regulation of excessive neuroinflammation is a promising therapeutical strategy to the treatment of demyelinated diseases.

Pyroptosis is a form of pro-inflammatory and lytic programmed necrosis mediated by gasdermins [6]. Pyroptosis is initiated by inflammasome activation, then followed by caspase-1 or caspase-11/4/5 activation which cleaves gasdermin D (GSDMD). The active N terminal separated from inactive C terminal of GSDMD form a gasdermin pore on plasma membrane of cells and lead to cell lysis [7], usually accompanied by IL-1β and interleukin-18 (IL-18) release [8]. Diverse cell types in CNS undergo pyroptosis in pathological conditions, including microglia/macrophages, neurons, oligodendrocytes and astrocytes [9]. Recent studies have found pyroptosis in multiple neurological diseases including multiple sclerosis (MS), Alzheimer’s disease (AD), stroke and traumatic brain injury (TBI) [10, 11]. Inhibition and deficiency of caspase-1 or nod-like receptor family, pyrin domain containing 3 (NLRP3) inflammasome have shown benefits in regulating inflammation and outcomes in AD, experimental autoimmune encephalomyelitis (EAE), TBI, brain ischemia and Parkinson disease (PD) [10–14]. NLRP3 inflammasome dysregulation has also been linked to severity of neuromyelitis optica spectrum disorders in patients [15]. These studies collectively suggest the important role of pyroptosis in neuroinflammation and demyelination.

Tregs is a subset of T cells characterized by CD4^+^CD25^+^Foxp3^+^ and occupy 10–15% of CD4^+^ T cells [16]. As the major immunosuppressive cells, Tregs play an essential role in maintaining homeostasis, regulating inflammatory responses and inhibiting autoimmune diseases [17]. Tregs may exert function via diverse anti-inflammatory mediators including interleukin-10 (IL-10), transforming growth factor-β (TGF-β) and interleukin-35 (IL-35) or negatively regulate target immune cells [18]. Currently, Tregs have been proved to benefit diverse CNS diseases, including ischemic stroke, intracerebral hemorrhage, TBI, spinal cord injury and autoimmune diseases [19–21]. Moreover, previous studies have suggested that Tregs may promote myelin repair in stroke and toxin-induced demyelination through osteopontin and CCN3 secretion, respectively [22, 23]. However, the roles of Tregs in pyroptosis and LPC-induced demyelination remain to be explored.

In present study, we established two-site LPC-induced demyelination model to explore the dynamic alterations of immune cells in lesion. Tregs were depleted in Foxp3-DTR/eGFP transgenic (DEREG) mice to investigate the role of Tregs in regulating myelin injury, inflammatory responses and pyroptosis in LPC-induced demyelination. Caspase-1 inhibitor VX765 was used to uncover the role of pyroptosis in myelin injury. The underlying regulatory mechanism by Tregs was explored by RNA-sequencing and verified by TLR4 inhibitor TAK-242.

## Material and methods

### Animals

All experiments were approved by the Institutional Animal Care and Use Committee of Tongji Medical College, Huazhong University of Science and Technology (Ethics approval number: TJH-202001002). Foxp3-DTR/eGFP mice (DEREG, male, 8–10 weeks old, #032050‐JAX, The Jackson Laboratory, USA) and C57BL/6 mice (WT, male, 8–10 weeks old, Biont, China) were used in the experiment. All animals were housed under standard laboratory conditions (22 ℃, 12-h/12-h dark–light cycle with food and water at libitum in the specific pathogen-free conditions). All animals were assigned randomly into sham-operation and LPC injection groups. Mice used were DEREG mice unless specifically stated with WT mice.

### Focal white matter demyelination by LPC injection

Two-site LPC injection was performed in accordance to previous study [24] with minor modifications. Briefly, mice were anesthetized with 2% isoflurane, and maintained with 1% isoflurane during LPC injection. Focal corpus callosum (CC) demyelination was induced by stereotaxic injection of 2 μl of 1% LPC (Sigma-Aldrich) in PBS with a 10 μl Hamilton syringe at the following sites relative to bregma: (1) 1.0 mm lateral, 0.8 mm anterior and 2.05 mm deep, (2) 1.0 mm lateral, 0.3 mm anterior and 1.9 mm deep. The rate of injection was 0.4 μl/min. The needle was kept for 10 min after injection and then pulled out slowly. The sham-operated mice suffered the same procedure except for receiving injection of PBS instead of LPC. During the surgery, mice were put on a heating pad to keep body temperature.

### Tregs depletion and agent administration

To deplete Tregs, DEREG mice were injected intraperitoneally with 1 μg DT (List Biological Laboratories, inc, USA) 24 h before LPC injection, then 0.5 μg DT every third day until 10 days after LPC injection. Control mice were treated with PBS injection. Flow cytometry in spleens and blood were used to confirm Tregs depletion.

In accordance with previous study [25], caspase-1 inhibitor VX765 (Selleck chemicals, USA) was intraperitoneally administrated at 50 mg/kg daily for pyroptosis inhibition in Tregs-depleted mice. And to study the role of TLR4 in pyroptosis, mice with Tregs abolition were injected with TLR4 inhibitor resatorvid (TAK-242, MedChemExpress, China) intraperitoneally at 3 mg/kg daily for 10 days according to a previous study [26].

### Flow cytometry

Single cell suspensions acquired from spleens and blood were first blocked with rat anti-CD16/32 (1:50, BD biosciences, Cat# 553,141) for 15 min at 4 ℃ and then incubated with CD4-APC (1:100, Biolegend, Cat# 100411) for 30 min at 4 ℃. Data were acquired on CytoFLEX3 (Beckman Coulter, USA) and analyzed with Flowjo V10 (TreeStar, Ashland, OR, USA).

### Tissue preparation

Mice were deeply anesthetized and killed at 10 days post-injection (dpi). All animals were transcardially perfused with ice-cold PBS. For immunofluorescent staining, the mice were then transcardially perfused with ice-cold 4% paraformaldehyde (PFA) (Servicebio, China). The brains were post-fixed in 4% PFA at 4 ℃ overnight and dehydrated in 30% (w/v) sucrose in PBS for 3 days. Then the brains were fast frozen in isopentane. The coronal sections of 12 μm were prepared with a freezing microtome (CryoStar NX50 OP, Thermo scientific, USA) and then stored at − 80 ℃. For western blotting, after PBS perfusion, the lesions in the CC were dissected carefully on ice under a microscope and stored at -80 ℃ quickly for later protein extraction.

### Immunofluorescence

The slices were washed in PBS and then permeabilized with 0.25% Triton-X100 (Solarbio life sciences, China) for 15 min at room temperature. After permeabilization, the brain slices were blocked in blocking buffer (QuickBlock™ Blocking Buffer for Immunol Staining, Beyotime, China) for 20 min at room temperature and followed by primary antibodies incubation. The following primary antibodies were used: rat anti-CD45 (1:200, Biolegend, Cat# 103101), rat anti-CD3 (1:200, Invitrogen, Cat# 14-0032-82); rabbit anti-Foxp3 (1:200, Cell Signaling Technology, Cat# 12653), rabbit anti-ionized calcium-binding adapter molecule 1 (Iba1) (1:400, Wako, Cat# 019-19741), rat anti-CD68 (1:400, Bio-Rad Laboratories, Cat# MCA1957), rabbit anti-NG2 (1:200, Millipore, Cat# AB5320), rabbit anti-GST-Pi (1:200, Medical and Biological Laboratories, Cat# 312), goat anti-Iba1 (1:200, Novus Biologicals, Cat# NB100-1028), rabbit anti-caspase-1 (1:50, Novus Biologicals, Cat# NBP1-45433), rabbit anti-GSDMD (1:100, Abcam, Cat# ab219800), rabbit anti-NLRP3 (1:40, Novus Biologicals, Cat# NBP2-12446), goat anti-IL-1β (1:100, R&D systems, Cat# AF-401-NA), mouse anti-GFAP (1:400, Cell Signaling Technology, Cat# 3670S), goat anti-Olig2 (1:40, R&D systems, Cat# AF2418-SP). After incubation with primary antibodies overnight at 4 ℃, the brain slices were washed in PBS for 10 min for 3 times and incubated with corresponding secondary antibodies. The following secondary antibodies from Jackson ImmunoResearch Laboratories were used: CY3-conjugated donkey anti-rabbit IgG (1:400, Cat# 711-166-152), Alexa Fluor 488-labeled donkey anti-rabbit IgG (1:200, cat# 711-545-152), CY3-conjugated donkey anti-goat IgG (1:200, Cat# 705-165-003), Alexa Fluor 488-labeled donkey anti-goat IgG (1:200, Cat# 705–545-147), Alexa Fluor 488-conjugated donkey anti-mouse IgG (1:200, Cat# 715-545-150). At last, the slices were washed, stained with 4,6-diamidino-2-phenylindole (DAPI) and mounted. Pictures were taken by fluorescent microscope (BX51, Olympus, Japan) and confocal microscopy (FV1200, Olympus, Japan). The positive area of Iba1 and CD68 were assessed by Image J (National Institutes of Health, USA). And double-immunopositive cells were counted manually by investigators blinded to the groups of the study. Three-dimensions reconstitution of microglia was performed with Imaris software (Bitplane, Switzerland).

### Western blot

Protein from the lesion was extracted with RIPA lysis buffer (Beyotime, China) containing PMSF, protease inhibitor cocktail and phosphatase inhibitor (all from Servicebio, China). BCA kit (Beyotime, China) was used to detect the protein concentration. Total protein (20 μg) was loaded on 10% sodium dodecyl sulfate–polyacrylamide gel electrophoresis (SDS-PAGE), and then transferred to nitrocellulose membranes (Boster Biological Technology, China). Membranes were blocked in 5% non-fat milk for 1 h at room temperature and incubated with the following primary antibodies: rat anti-MBP (1:500, Millipore, Cat# MAB386), rabbit anti-NF-H (1:1000, Proteintech, Cat# 18934-1-ap), rabbit anti-NF-M (1:1000, Proteintech, Cat# 25805-1-ap), rabbit anti-NF-L (1:1000, Proteintech, Cat# 25805-1-ap), rabbit anti-caspase-1 (1:1000, abclonal, Cat# A0964), mouse anti-GSDMD (1:1000, Santa Cruz, Cat# sc-393581), mouse anti-cleaved caspase-1 (1:1000, Santa Cruz, Cat# sc-398715), rabbit anti-cleaved GSDMD (1:1000, Cell Signaling Technology, Cat# 10137S), rabbit anti-NLRP3 (1:1000, Cell Signaling Technology, Cat# 15101), rabbit anti-nod-like receptor family, CARD domain containing 4 (NLRC4) (1:1000, abclonal, Cat# A7382), rabbit anti-MyD88 (1:1000, Abcam, Cat# ab219413), rabbit anti-NF-κB p65 (1:1000, Cell Signaling Technology, Cat# 8242T), rabbit anti-Phospho-NF-κB P65 (1:1000, Cell Signaling Technology, Cat# 3033T), rabbit anti-β-actin (1:1000, Servicebio, Cat# GB11001). After incubation for 16–18 h at 4 ℃, the membranes were incubated with corresponding horseradish peroxidase-conjugated goat anti-rabbit (Cat# 111-035-003), goat anti-mouse (Cat# 115-035-003) and goat anti-rat (Cat# 112-035-003) secondary antibodies (1:5000) for 1 h at room temperature. Secondary antibodies were from Jackson ImmunoResearch. Then the membranes were visualized with enhanced chemiluminescent kits (Advansta, USA) and Gelview 6000 pro system (BLT, China). The integrated optical densities (OD) were collected and analyzed with Image J. The expressions of protein were normalized to internal controls of β-actin. We have provided the raw images of western blot in Additional file 2.

### qRT-PCR

The total RNA of demyelinated tissue in CC was extracted with Trizol reagent (Invitrogen, USA) at 10 dpi and immediately reversely transcribed into cDNA with PrimerScript^TM^ RT Mater Mix (Takara, Japan). Then qRT-PCR was performed on Real-Time PCR system (BioRad, USA) with SYBR Green PCR Master Mix (Yeasen, China) and specific primers. Primers used are supplied in Supplementary Table 1. All primers were from Sangon biotech, China. The cycle times were normalized to corresponding GAPDH as an internal control and results were shown as fold changes compared with sham group.

### LFB staining

At 10 dpi, LFB staining was performed to detect demyelinated lesions in CC according to a previous study [27]. Briefly, after washed in PBS, brain slices were incubated in pre-warmed 0.1% LFB staining buffer (Servicebio, China) at 60 ℃ for 6–8 h, then cooled and differentiated in Li_2_CO_3_ for 2 min. When differentiation was completed, slices were successively dehydrated in 75%, 95% and 100% alcohol and waiting for air-dried. Then slices were mounted by neutral gum (Baso, China). Photos were taken with an optical microscope (BX51, Olympus, Japan).

### Neurobehavioral assessment

#### Morris water maze

Morris water maze was performed at 5 dpi and repeated daily to 10 dpi to assess spatial cognitive functions according to previous studies [28, 29]. A tank with diameter of 120 cm filled with water, with a platform hidden 1 cm below the surface of water was used. Water temperature was kept between 22 ℃ and 25 ℃. Mice were subjected to swimming for 1 min to find the hidden platform and learning for 30 s on the platform, repeating 4 times daily with different starting quadrants. Mice were led to platform if they failed to find it. ANY-maze software (Stoelting, CO, USA) was used to record time spent to find platform. The learning procedure was repeated for 5 days. At 10 dpi, the platform was removed and mice underwent a probe trial by swimming freely in the tank for 1 min, the percent time spent in target quadrant was recorded by ANY-maze software.

#### Novel object recognition

Working memory was evaluated by novel object recognition as described previously [30]. Briefly, mice explored freely in a 40 × 40 × 40 cm box for 5 min on the first day to get familiar to the environment. On the second day, mice explored for 10 min with two identical cubes at symmetrical places inside the box. And on the next day, a new object with different color and shape replaced one of the cubes. During the test phase, the exploration time for mice was 5 min. In novel object recognition test, ANY-maze software was used to record the exploration time spent on new (N) and familiar (F) objects. The preference for new and familiar objects was calculated by discrimination index (DI): $DI=(N-F)/(N+F)$.

### RNA-sequencing and analysis

All samples were tested using the DNBSEQ platform, with an average yield of 6.75G data per sample. Oligo dT were used to enrich the mRNA. After RNA fragmentation, cDNA synthesis and purification, sequencing was performed on DNBSEQ platform. Raw reads were filtrated by software SOAPnuke as follows: (1) remove the reads containing the adaptor (adaptor pollution); (2) remove reads whose N content is greater than 5%; (3) remove low-quality reads (reads with bases with a quality score less than 15 as the proportion of total bases in the reads that are greater than 20%). After quality control, the filtered clean reads were aligned to the GENCODE GRCm39 mouse genome as the reference sequence with Hierarchical Indexing for Spliced Alignment of Transcripts (HISAT) software. All samples passed the quality control. Differentially expressed genes (DEGs) were defined when *Q* < 0.05 and |log2FC|> 1. KEGG pathway enrichment analysis, GO enrichment analysis, protein–protein interaction (PPI) network, gene set enrichment analysis (GSEA) and key driver analysis (KDA) were then performed among the screened DEGs.

### Statistical analysis

All data were presented as mean ± standard deviation (SD) and were analyzed by GraphPad Prism 8. For statistical analysis, Shapiro–Wilk test was first used to detect normal distribution of data, then two-tailed Student’s t-test for comparison between two groups, one-way or two-way analysis of variance (ANOVA) with Tukey’s or Bonferroni’s post hoc test for multiple comparisons were applied. *P* < 0.05 was considered statistically significant.

## Results

### Tregs infiltrate in demyelinated lesions and depletion of Tregs aggravates lymphocytes infiltration and microgliosis

**Fig. 1:**
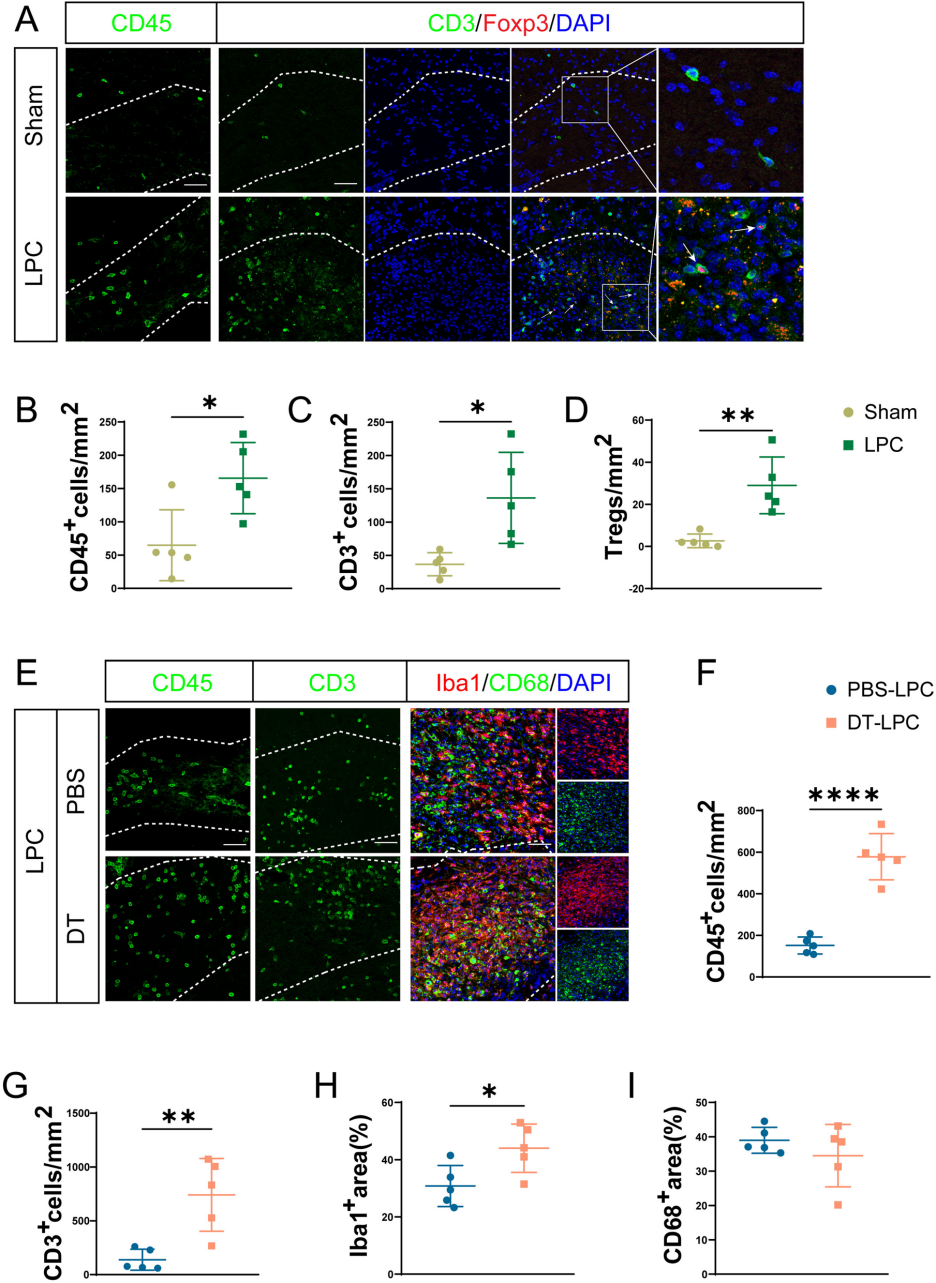
The dynamic alterations of Tregs after LPC injection and depletion of Tregs aggravates lymphocytes infiltration as well as microgliosis. **A** Representative images of infiltration of CD45^+^ leukocytes and CD3^+^Foxp3^+^ Tregs in CC at 10 dpi of sham and LPC group. CC in sham group and lesions in LPC group were indicated by dashed line. Enlarged images of Tregs infiltration were shown in right panel, and Tregs were indicated by white arrows. Scale bar = 50 μm. **B**–**D** Quantification of CD45^+^ leukocytes (**B**), CD3^+^ T lymphocytes (**C**) and Tregs (**D**) in CC at 10 dpi of sham and LPC group. **E** Representative immunofluorescent images of CD45^+^ leukocytes, CD3^+^ T lymphocytes infiltration and staining of Iba1 and CD68 in CC at 10 dpi in PBS-treated and DT-treated LPC group were shown, demyelinated lesions were indicated by dashed line, scale bar = 50 μm. **F**, **G** Statistical analysis of CD45^+^ leukocytes (**F**) and CD3^+^ T lymphocytes (**G**) in lesions of LPC group treated by PBS and DT. **H**, **I** Quantification of Iba1^+^ area (**H**) and CD68^+^ area (**I**) in lesions of LPC group treated by PBS and DT. Two-tailed Student’s *t*-test was used, *n* = 5/group. *P < 0.05, **p < 0.01, ***p < 0.001, ****p < 0.0001

According to previous studies [24, 29], two-point injection of LPC induced demyelination and striking microglia activation at 10 dpi. Therefore, we investigated lymphocytes infiltration and neuroinflammation at 10 dpi in this study. As shown in Fig. 1A, PBS injection in sham group led to minor CD45^+^ leukocytes and CD3^+^ T cells infiltration which mainly accumulated surrounding the needle hole, suggesting the needle puncture induced blood–brain barrier (BBB) breakdown. Meanwhile, CD3^+^Foxp3^+^ Tregs were rarely observed after PBS injection (Fig. 1A). Compared with PBS injection, abundant CD45^+^ leucocytes and CD3^+^ T cells infiltrated and accumulated within the demyelinated lesion induced by LPC, with Tregs scattering inside lesion at 10 dpi (Fig. 1A–D).

To further explore the role of Tregs in the process of LPC-induced demyelination, DEREG mice were intraperitoneally administrated with DT to deplete Tregs. Flow cytometry showed that DT significantly decreased the proportion of Tregs both in blood and spleen (Additional file 1: Fig. S1A-C). Then PBS or DT-treated DEREG mice were subjected to LPC injection. We found that depletion of Tregs significantly aggravated CD45^+^ leucocytes and CD3^+^ T cells infiltration (Fig. 1E–G). Moreover, by immunolabelling of Iba1 and CD68, we observed increased area of Iba1 positive cells in Tregs-depleted mice compared with PBS-LPC group (Fig. 1E, H). The CD68^+^ area, a marker of microglia phagocytosis, were comparable in these two groups (Fig. 1E, I). These data indicate that Tregs infiltrate in demyelinated lesion and alleviate microgliosis as well as infiltration of adaptive immune cells in LPC-induced demyelination.

### Tregs depletion exacerbates LPC-induced demyelination

**Fig. 2:**
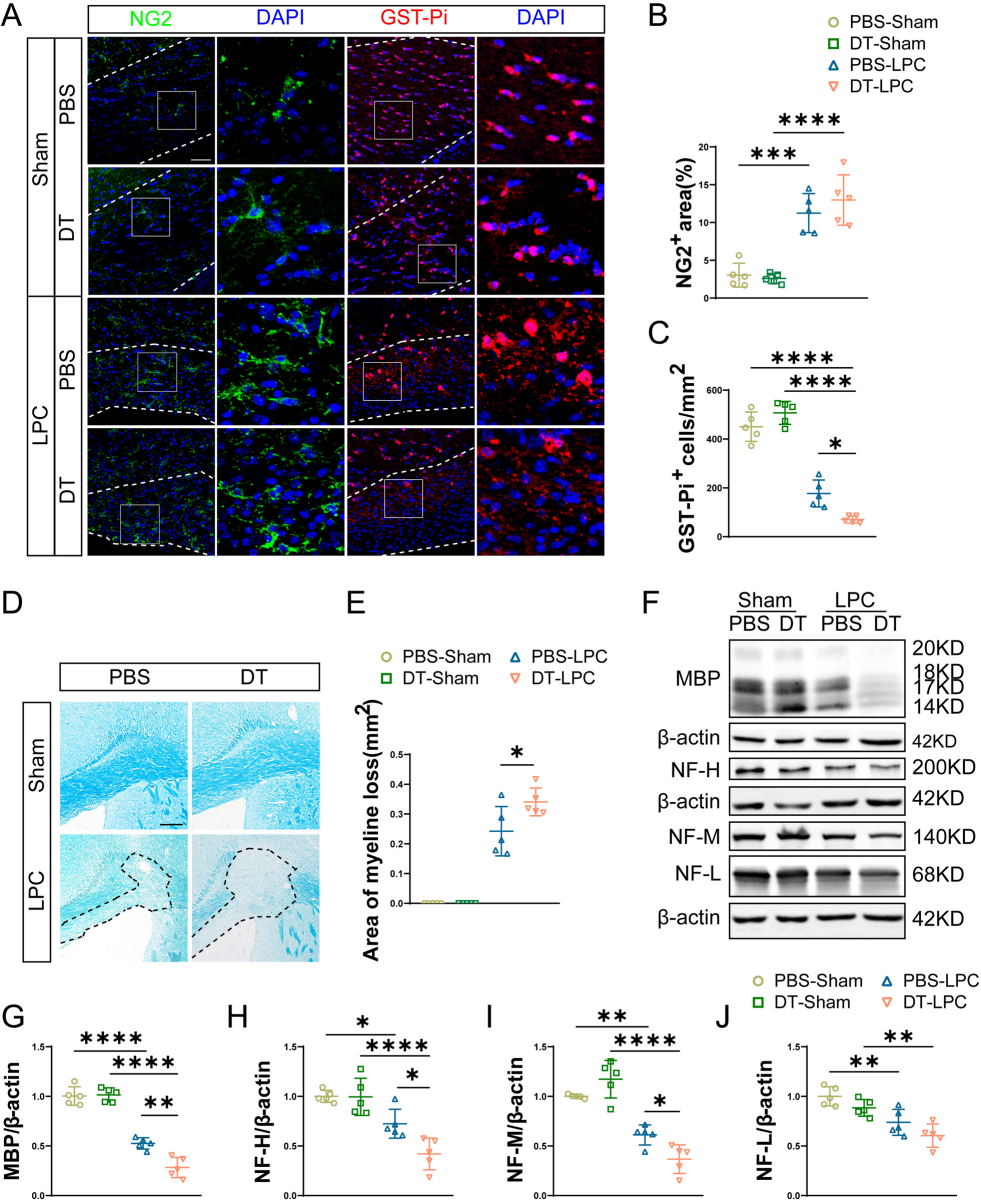
Depletion of Tregs exacerbates myelin injury in LPC-induced demyelination. **A** Representative confocal images of NG2^+^ OPCs and GST-Pi^+^ mature oligodendrocytes in sham group and LPC-demyelination group treated with PBS and DT, respectively. CC in sham group and lesions in LPC group were indicated by dashed line. Enlarged views of square area were shown. Scale bar = 50 μm. **B** Quantification of NG2 positive area in lesions of sham and LPC group treated with PBS and DT. **C** Statistical analysis of GST-Pi^+^ mature oligodendrocytes in lesions in PBS and DT-treated sham or LPC-induced demyelinated mice. **D**, **E** Representative LFB staining of CC in different groups (**D**) and quantification of demyelinated areas (**E**). Demyelinated areas were indicated by dashed black lines. Scale bar = 200 μm. **F** Representative images of western blot of MBP, NF-H, NF-M, NF-L and β-actin among groups. **G-J** Quantification of protein expressions of MBP (**G**), NF-H (**H**), NF-M (**I**) and NF-L (**J**) in sham and LPC-demyelination group treated by PBS and DT. *N* = 5/group. Two-way ANOVA with Tukey’s post hoc test was used. *P < 0.05, **p < 0.01, ***p < 0.001, ****p < 0.0001

Next, we studied the effects of Tregs on LPC-induced demyelination by depletion of Tregs. After LPC injection, immunofluorescence staining displayed an increase in NG2 expression and a decrease in GST-Pi^+^ mature oligodendrocytes compared with sham animals (Fig. 2A–C), suggesting proliferation of oligodendrocyte progenitor cells (OPCs) (NG2^+^) and loss of mature oligodendrocytes (GST-Pi^+^). Compared with PBS-LPC group, depletion of Tregs further aggravated mature oligodendrocytes loss (Fig. 2A, C) but had no influence on OPCs proliferation as evidenced by NG2^+^ area (Fig. 2A, B). We next applied LFB staining and western blot to assess severity of myelin injury. By LFB staining, we observed larger lesions in Tregs-depleted mice compared with PBS-treated mice after LPC administration (Fig. 2D, E). In consistent with immunofluorescent and LFB staining, western blot analysis showed lower expression of MBP in LPC group compared with sham group. Depletion of Tregs further reduced the protein expression of MBP compared with PBS-LPC group (Fig. 2F, G). Meanwhile, axonal damage was observed as evidenced by the lower expressions of NF-H, NF-M and NF-L after LPC injection (Fig. 2F, H–J). Moreover, depletion of Tregs triggered decreased expressions of NF-H and NF-M compared with PBS-LPC group (Fig. 2F, H, I). Taken together, our results showed exacerbated myelin injury by Tregs depletion in LPC-demyelination model.

### Tregs regulate inflammatory responses and pyroptosis in LPC-induced demyelination

**Fig. 3:**
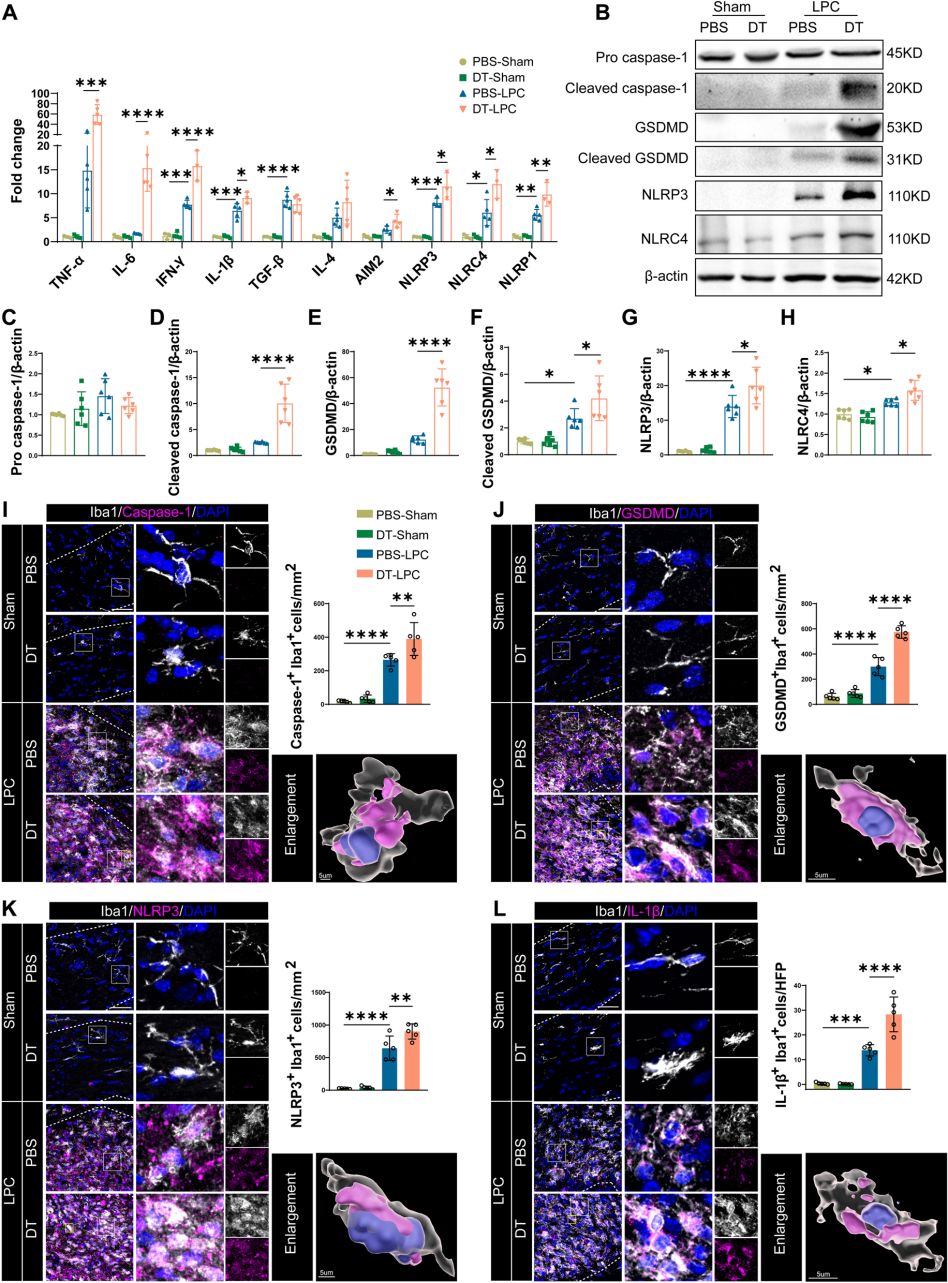
Tregs depletion aggravates inflammatory responses and pyroptosis of microglia in LPC-induced demyelination. **A** Expressions of mRNA of inflammatory cytokines and inflammasomes in different groups. *N* = 3–5 per group. Two-way ANOVA followed by Bonferroni’s test was applied. **B**–**H** Representative images of western blot for pyroptosis-related proteins in sham and LPC group treated with PBS and DT (B), and corresponding statistical analysis of protein expressions (**C**–**H**). *N* = 6/group. **I**–**L** Representative confocal double-labeling images of Iba1with caspase-1 (**I**), GSDMD (**J**), NLRP3 (**K**) and IL-1β (**L**) in CC and corresponding quantifications of double-positive cells among different groups. CC in sham group and lesions in LPC group were indicated by dashed line. Magnified images were shown and enlarged 3D views were recontributed by Imaris. Scale bars were 50 μm in confocal images and 5 μm in 3D images, respectively. N = 5/group. Two-way ANOVA with Tukey’s post hoc test was used. *P < 0.05, **p < 0.01, ***p < 0.001, ****p < 0.0001

Previous studies have proved that pyroptosis occurs in multiple inflammatory and neurodegenerative diseases including MS, amyotrophic lateral sclerosis (ALS), AD and PD [10, 14]. To explore pyroptosis in our model of LPC-induced demyelination, we first performed qRT-PCR to examine the transcriptional expressions of inflammatory cytokines and pyroptosis-related proteins in demyelinated lesions. As shown in Fig. 3A, mRNA expressions of pro-inflammatory cytokines including TNF-α, interleukin-6 (IL-6), IFN-γ and IL-1β were upregulated or in a trend to increase after LPC injection. Remarkably, Tregs depletion by DT further augmented their expressions compared with PBS-LPC group (Fig. 3A). Similarly, increased mRNA expressions of inflammasomes NLRP3, NLRC4 and nod-like receptor family, pyrin domain containing 1 (NLRP1) were observed in LPC-treated mice compared with sham group, which were further increased after Tregs depletion (Fig. 3A). In addition, the results of western blot further validated findings of qRT-PCR. In LPC-induced demyelinated mice, increased protein expressions of cleaved GSDMD, NLRP3 and NLRC4 versus sham group were observed, while cleaved caspase-1 and GSDMD appeared in trends to upregulate in demyelinated mice (Fig. 3B, D–H). Moreover, depletion of Tregs triggered higher protein expressions of GSDMD, cleaved caspase-1, cleaved GSDMD, NLRP3 and NLRC4 compared with PBS-LPC group (Fig. 3B, D–H). In contrast, non-active pro caspase-1 showed comparable protein levels among different groups (Fig. 3B, C).

We next investigated the main cell type that underwent pyroptosis in our LPC-induced demyelination by co-staining of pyroptosis markers with different cell markers. Rare pyroptosis was observed in CC in sham animals as shown by immunofluorescence (Fig. 3I–L). By co-staining of pyroptosis executant GSDMD and typical cell markers, we identified that LPC induced severe pyroptosis in microglia but rarely in astrocytes and oligodendrocytes in LPC-induced demyelination (Fig. 3J, Additional file 1: Fig. S2A, B). Additional double-labeling of caspase-1, NLRP3, IL-1β with Iba1 further confirmed abundant pyroptosis in microglia in our model, and 3D reconstruction further showed expressions of caspase-1, GSDMD, NLRP3 and IL-1β in microglial cytoplasm (Fig. 3I–L). Consistent with above results of western blot, immunofluorescence exhibited an increase in microglial pyroptosis after Tregs depletion (Fig. 3I–L). Here we found pyroptosis of microglia in LPC-induced demyelination, and revealed that depletion of Tregs exacerbated neuroinflammation and microglial pyroptosis in LPC-induced demyelination.

### Pyroptosis inhibitor restores myelin injury and memory impairment aggravated by depletion of Tregs after LPC-induced demyelination

**Fig. 4:**
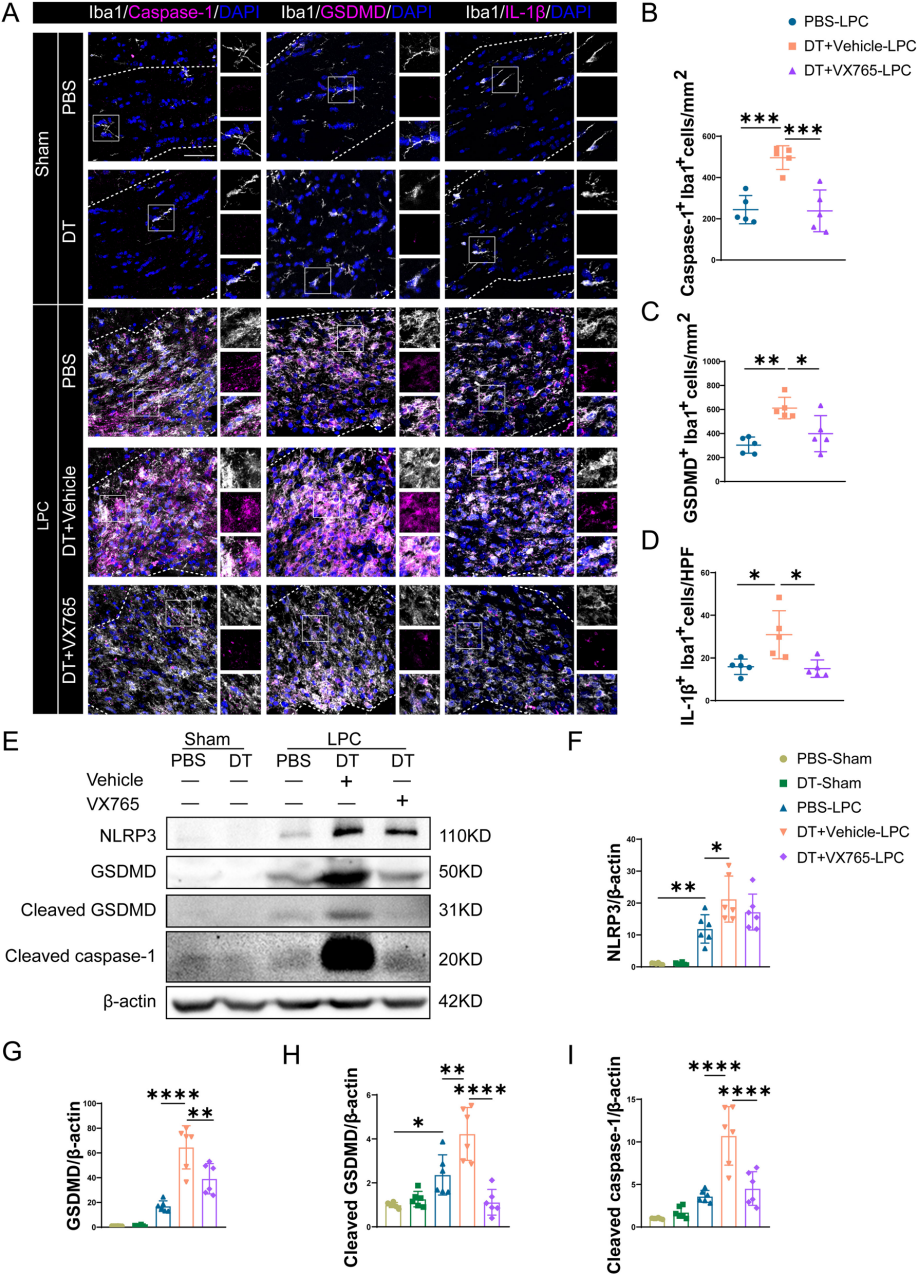
Caspase-1 inhibitor VX765 significantly decreases pyroptosis of microglia which is enhanced by Tregs ablation. **A** Confocal images of Iba1 co-staining with caspase-1, GSDMD and IL-1β in sham and LPC group treated by PBS, DT plus vehicle and DT plus VX765 were shown, CC in sham group and lesions in LPC group were indicated by dashed line, magnified views were displayed. Scale bar = 50 μm. **B**–**D** Caspase-1-positive microglia per mm^2^ (**B**), GSDMD-positive microglia per mm^2^ (**C**) and IL-1β-positive microglia per high-power field (HPF) (**D**) in lesions were quantified. *N* = 5 for each group. **E**–**I** Representative images for western blot of pyroptosis-related proteins NLRP3, GSDMD, cleaved GSDMD and cleaved caspase-1 in sham and LPC-demyelination group treated by PBS, DT with vehicle and DT with VX765 (**E**), statistical analyses of corresponding protein expressions were shown in F-I, respectively. *N* = 6/group. One-way ANOVA followed by Tukey’s post hoc test was used. *P < 0.05, **p < 0.01, ***p < 0.001, ****p < 0.0001

Since Tregs depletion triggered both exacerbated pyroptosis and aggravated myelin injury, we next investigated whether pyroptosis is involved in the effects of Tregs depletion on myelin injury and inhibiting pyroptosis affords protection against myelin injury induced by LPC. We applied caspase-1 inhibitor VX765 to prohibit microglial pyroptosis. First, immunofluorescent co-staining of caspase-1, GSDMD, IL-1β and Iba1 showed that VX765 significantly reduced microglial pyroptosis in Tregs-depleted mice compared to DT + vehicle-LPC group (Fig. 4A–D). And western blot exhibited consistent results. In LPC group, protein expressions of GSDMD, cleaved GSDMD and cleaved caspase-1 were all inhibited by VX765 compared with Tregs-depleted vehicle group (Fig. 4E, G–I). Meanwhile, protein expressions of NLRP3 in VX765 group were comparable to vehicle group (Fig. 4E, F). These results confirmed that capase-1 inhibitor VX765 successfully prevented pyroptosis in LPC-induced demyelination.

**Fig. 5:**
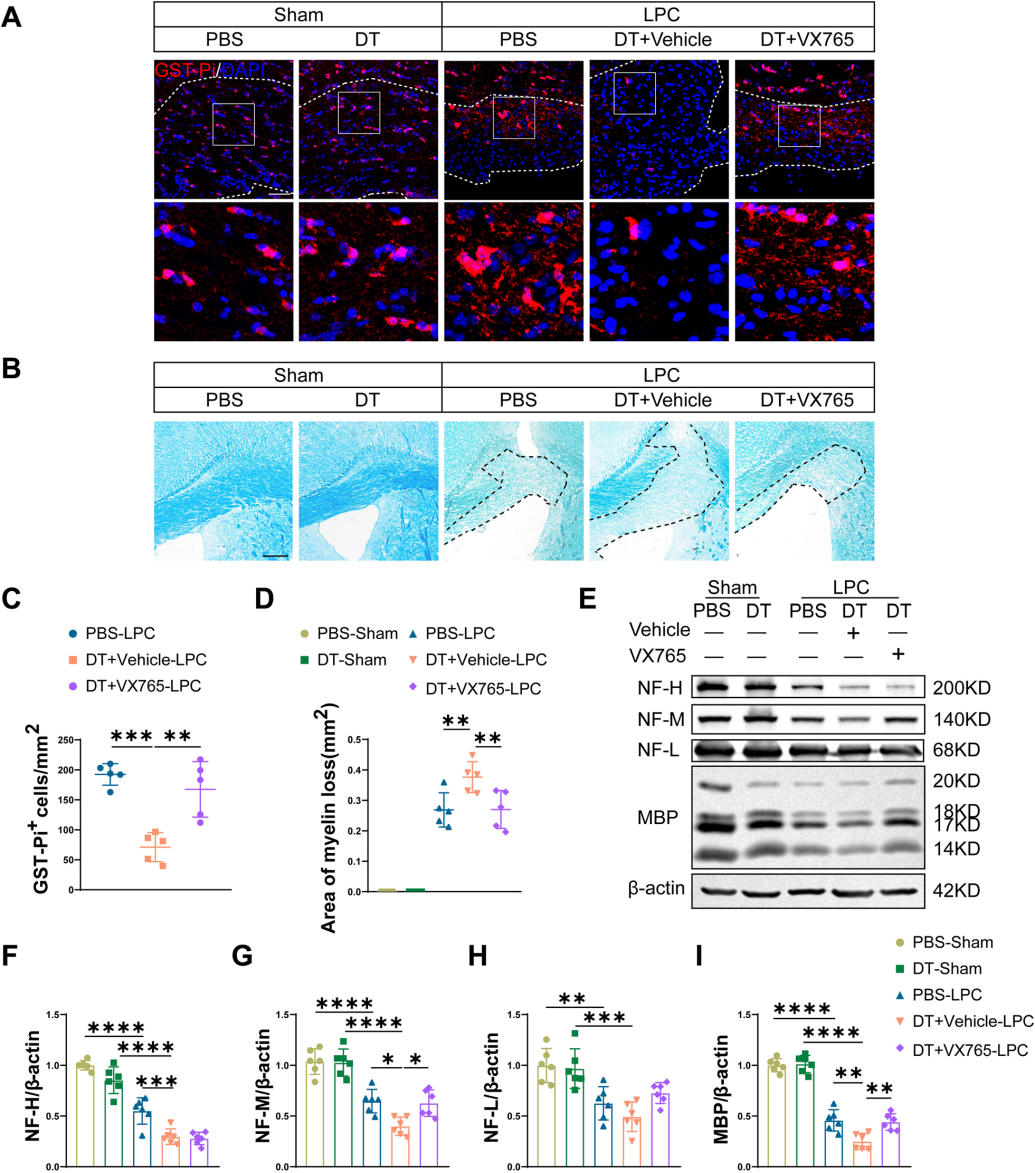
Caspase-1 inhibitor VX765 partly reverses myelin injury aggravated by Tregs depletion. **A** Representative confocal images of GST-Pi^+^ mature oligodendrocytes in lesions in sham and LPC group with different treatments. CC in sham group and lesions in LPC group were outlined by dashed white lines, and enlarged images were displayed in the down panel. Scale bar = 50 μm. **B** LFB staining of CC was shown among groups. Demyelinated areas were indicated by dashed black lines. Scale bar = 200 μm. **C** Quantification of GST-Pi^+^ mature oligodendrocytes per mm^2^ within lesions in LPC-demyelinated mice treated by PBS, DT + vehicle and DT + VX765. *N* = 5/group. **D** Quantification of demyelinated areas of LFB staining. *N* = 5/group.** E-I** Western blot of NF-H, NF-M, NF-L and MBP in sham and LPC group treated by PBS, DT plus vehicle and DT plus VX765 (**E**), corresponding statistical analysis for protein expressions of NF-H (**F**), NF-M (**G**), NF-L (**H**) and MBP (**I**) were shown. *N* = 6 for each group. One-way ANOVA with Tukey’s test was applied. *P < 0.05, **p < 0.01, ***p < 0.001, ****p < 0.0001

Next, we explored the severity of myelin injury after VX765 supplementation. As presented in Fig. 5A, C, VX765 treatment rescued the decrease in mature oligodendrocytes in Tregs-depleted mice. LFB staining also showed smaller lesion size in VX765-treated group compared with vehicle-treated group in mice with Tregs depletion (Fig. 5B, D). In addition, western blot showed higher protein expressions of both MBP and NF-M in mice treated with VX765 versus with vehicle in Tregs-depleted mice (Fig. 5E, G, I). However, protein expressions of NF-H and NF-L showed no differences between interventions by VX765 and vehicle (Fig. 5E, F, H).

**Fig. 6:**
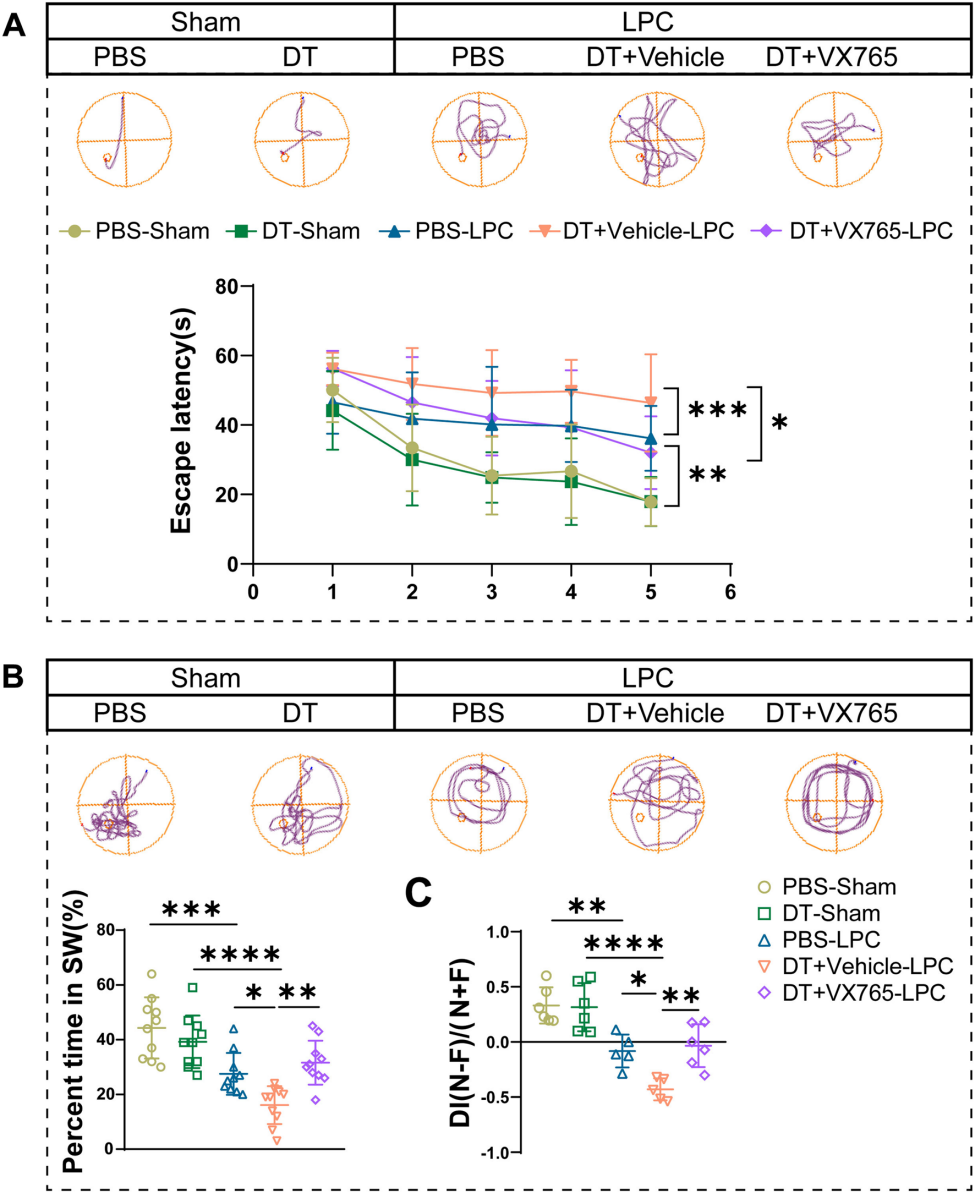
Caspase-1 inhibitor VX765 protects against cognitive impairment in LPC-induced demyelination. **A** In Morris water maze experiment, escape latency of mice during acquisition test from 5 to 9 dpi was recorded, representative tracks at 9 dpi were displayed. *N* = 10/group. Two-way ANOVA followed by Tukey’s test. **B** Statistical analysis of percent time spent in the target quadrant during the probe trial among groups. And representative tracks in different groups were displayed. *N* = 10/group. One-way ANOVA with Tukey’s post hoc test. **C** Novel object recognition test was performed from 8 to 10 dpi. DI was calculated to compare the preference for novel objects in different groups. *N* = 5–6, one-way ANOVA with Bonferroni’s test was applied. *P < 0.05, **p < 0.01, ***p < 0.001, ****p < 0.0001

Then Morris water maze and novel object recognition tests were conducted to explore the effects of Tregs and pyroptosis on spatial memory and working memory impairment induced by demyelination. First, LPC induced spatial memory deficit in mice, as evidenced by more time to find the hidden platform in learning process and less time spent in target area in probe trial in demyelinated mice (Fig. 6A, B), and depletion of Tregs in mice induced longer escape latency and decreased percent time in target area in comparison with mice in PBS-LPC group (Fig. 6A, B). In contrast, inhibition of pyroptosis by VX765 in Tregs-depleted mice rescued the longer escape latency caused by Tregs depletion. (Fig. 6A). And in probe trial, VX765 treatment in Tregs-depleted mice significantly increased time of mice hanging in target quadrant compared to Tregs-depleted mice treated with vehicle (Fig. 6B). Moreover, the novel object recognition test showed similar results. Demyelinated mice in LPC group presented less preference for novel objects compared with sham group. In mice with Tregs depletion, their interest in new objects further lost compared with PBS-treated demyelinated mice, which was reversed by VX765 treatment in Tregs-depleted mice (Fig. 6C). In conclusion, we found that inhibition of pyroptosis by VX765 in Tregs-depleted mice reversed myelin loss and cognitive defects aggravated by Tregs ablation.

### TLR4/MyD88/NF-κB pathway is involved in regulating pyroptosis by Tregs in LPC-induced demyelination

**Fig. 7:**
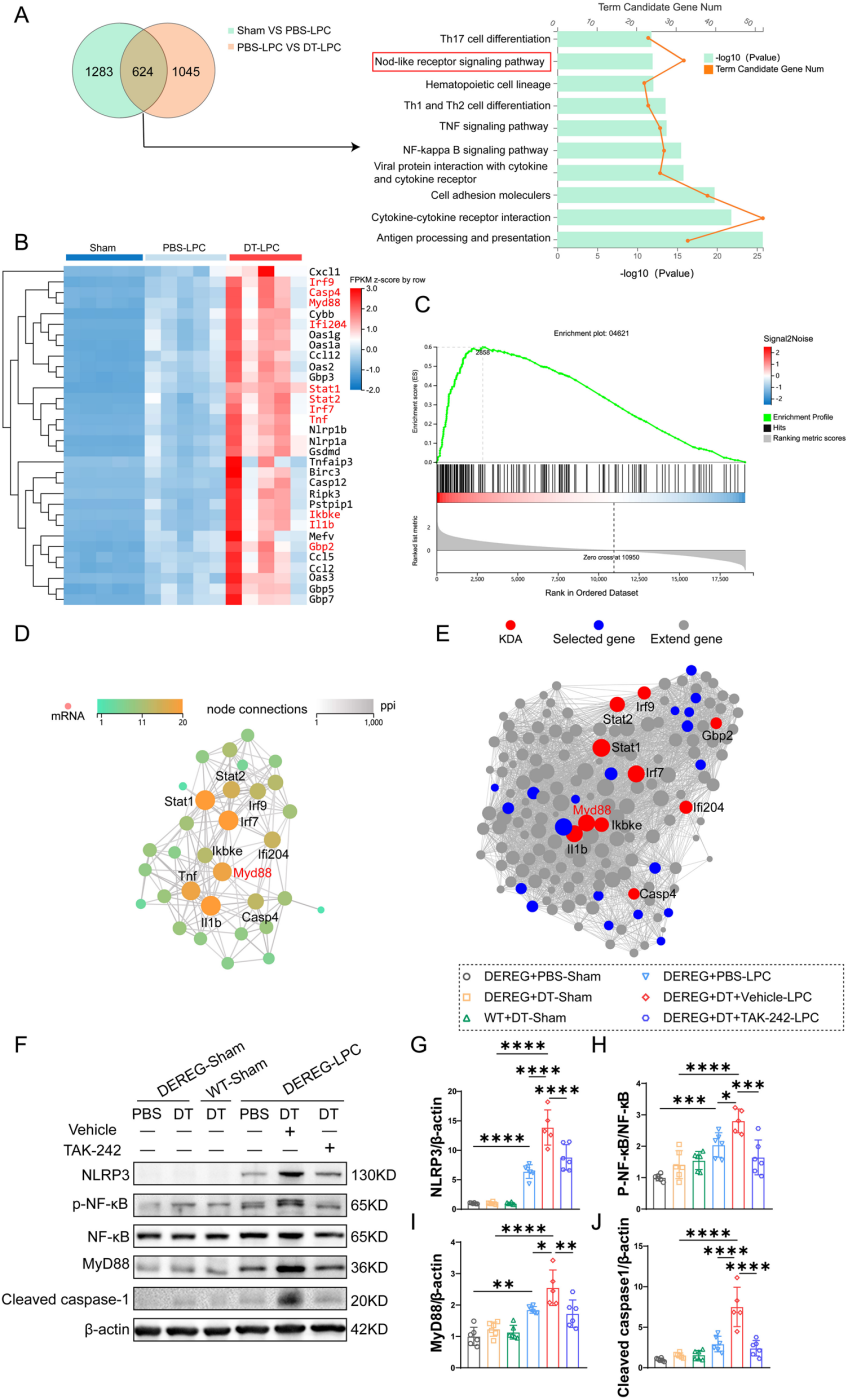
RNA-sequencing analysis reveals that TLR4/MyD88 play central roles in pyroptosis exacerbated by Tregs depletion in LPC-induced demyelination. **A** Venn diagram of DEGs between Sham VS PBS-LPC and PBS-LPC VS DT-LPC. KEGG pathway enrichment analysis of common DEGs was shown in the right panel. **B** Heatmap of hierarchical clustering analysis of DEGs involved in Nod-like receptor signaling pathway. DEGs appeared in **D** and **E** were marked in red. **C** GSEA analysis of Nod-like receptor signaling pathway. **D**, **E** PPI (**D**) and KDA (**E**) analysis of Nod-like receptor signaling pathway were shown, with the top ten DEGs indicated. For RNA-sequencing analysis, n = 5 replicates per group. **F**–**J** Representative images of western blot of NLRP3, p-NF-κB, NF-κB, MyD88 and cleaved caspase-1 (**F**), corresponding statistical analysis for protein expressions of NLRP3 (**G**), p-NF-κB/NF-κB (**H**), MyD88 (**I**) and cleaved caspase-1 (**J**) were shown. *N* = 5–6/group, one-way ANOVA with Bonferroni’s test was applied. *P < 0.05, **p < 0.01, ***p < 0.001, ****p < 0.0001

To explore the possible molecular mechanism underlying pyroptosis regulation by Tregs, we performed RNA-sequencing in sham, PBS-LPC and DT-LPC groups. Significant differences in gene expressions among these three groups were revealed by volcano plots (Additional file 1: Fig. S3A, B) and expression heat map (Additional file 1: Fig. S3C). As shown in Fig. 7A, 1907 DEGs between sham and PBS-LPC group and 1669 DEGs between PBS-LPC and DT-LPC group were found, while 624 shared DEGs were identified. The 624 shared DEGs were then analyzed by KEGG pathway and GO enrichment analysis. The GO enrichment analysis showed the top affected pathways, suggesting that DEGs were mainly related to immune system and inflammatory response (Additional file 1: Fig. S3D), and KEGG pathway enrichment analysis revealed Nod-like receptor signaling pathway as an important pathway regulated by Tregs (Fig. 7A), which is tightly involved in pyroptosis [31, 32]. Therefore, it is conceivable that depletion of Tregs exacerbated microglial pyroptosis in LPC-induced demyelination since our RNA-seq suggested that Nod-like receptor signaling was one of the top pathways regulated by Tregs. Next, we performed expression heat map of genes involved in Nod-like receptor signaling pathway. As shown in Fig. 7B, we observed elevated gene expressions in PBS-LPC group compared to sham group, while depletion of Tregs further upregulated their expressions. The GSEA also revealed that the Nod-like receptor signaling pathway was significantly enriched in DT-treated demyelinated mice (Fig. 7C). Thus, we focused on Nod-like receptor signaling pathway and performed PPI and KDA analysis, the results suggested MyD88 as one of the central molecules to regulate Nod-like receptor signaling pathway and pyroptosis in our LPC-demyelination model (Fig. 7D, E). Next, to distinguish Tregs depletion-mediated effects from DT-mediated toxicity, we performed qRT-PCR containing DT-treated WT sham group to verify the top ten key genes of KDA in RNA-seq. As shown in Additional file 1: Fig. S4, our results of qRT-PCR confirmed upregulation of key genes in Nod-like receptor signaling pathway after LPC-induced demyelination. Moreover, these genes were further upregulated after Tregs depletion. We found that DT treatment alone only induced increased expression of Stat1 while had no influence on other key genes involved in Nod-like receptor signaling pathway.

MyD88 is an adaptor of multiple Toll-like receptors (TLRs), in which TLR4 is mostly studied. In CNS, TLR4 is mainly expressed in microglia [33, 34]. TLR4 can recognizes pathogen-associated molecular patterns (PAMPs) and damage-associated molecular patterns (DAMPs), then activates NF-κB and MAPKs in a MyD88-dependent way, leading to expressions of pro-inflammatory cytokines [35, 36]. Therefore, we applied a TLR4 inhibitor TAK-242 to test whether Tregs regulated pyroptosis via TLR4/MyD88. Firstly, we found that DT treatment alone had no effects on TLR4/MyD88/NF-κB pathway and pyroptosis in LPC-induced demyelination as evidenced by comparable expressions of Myd88, p-NF-κB/NF-κB ratio, NLRP3 and cleaved caspase-1 in three sham groups (DEREG + PBS, DEREG + DT and WT + DT) (Fig. 7F–J). As shown in Fig. 7F, H, I, expression of MyD88 and p-NF-κB/NF-κB ratio were significantly increased after LPC-demyelination, and Tregs depletion induced higher protein expression of MyD88 and evaluated ratio of p-NF-κB/NF-κB than PBS-LPC group, indicating that TLR4/MyD88/NF-κB pathway was further activated in Tregs-depleted LPC group, which is consistent with our results of RNA-sequencing. TAK-242 treatment successfully reversed the overactivated TLR4/MyD88/NF-κB pathway in Tregs-depleted mice as evidenced by decreased expression of MyD88 and ratio of p-NF-κB/NF-κB compared with DT + vehicle-LPC group (Fig. 7F, H, I). Moreover, while ablation of Tregs aggravated pyroptosis-related protein expressions of NLRP3 and cleaved caspase-1, they were reversed by TAK-242 treatment (Fig. 7F, G, J). Taken together, our results indicate that Tregs regulate pyroptosis via TLR4/MyD88/NF-κB pathway in LPC-induced demyelination.

## Discussion

**Fig. 8:**
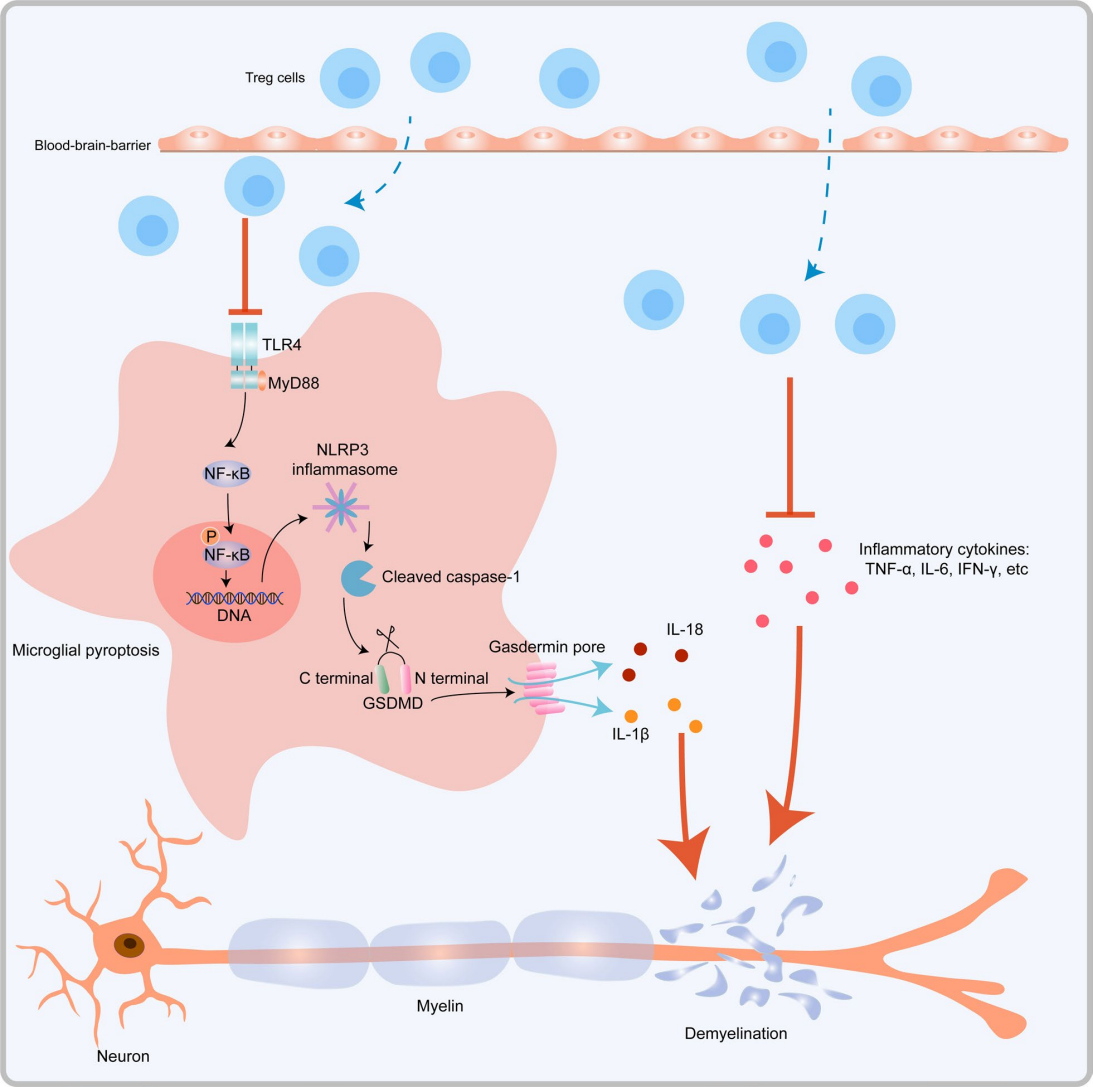
The schematic diagram illustrating how Tregs protect against myelin injury by alleviating pyroptosis and neuroinflammation in demyelination induced by LPC. LPC-demyelination induces pyroptosis in microglia and inflammatory responses in CNS. Mechanically, Tregs alleviate pyroptosis by inhibiting TLR4/MyD88 and downstream phosphorylation of NF-κB, which inhibit NLRP3 inflammasome and subsequent pyroptosis. In addition, Tregs could regulate neuroinflammation by suppressing inflammatory cytokines including TNF-α, IL-6, IFN-γ and IL-1β

In this study, Tregs depletion induced aggravated inflammatory responses and pyroptosis in microglia, accompanied by exacerbated myelin injury as well as cognitive defects in LPC-induced demyelination. In contrast, caspase-1 inhibitor VX765 treatment in Tregs-depleted mice successfully reversed increased microglial pyroptosis and restored myelin injury as well as cognitive defects aggravated by Tregs depletion. RNA-sequencing and TLR4 inhibitor TAK-242 treatment further verified that TLR4/MyD88/NF-κB pathway played a crucial role in pyroptosis regulated by Tregs. In summary, we substantiated that Tregs depletion aggravated myelin injury by exacerbating pyroptosis of microglia via TLR4/MyD88/NF-κB pathway (Fig. 8).

Tregs protect against diverse CNS diseases ascribed by limiting excessive neuroinflammation and pro-repair ability. Accumulating studies have found that Tregs regulate in situ glia activation, gliosis, infiltration and phenotypes of immune cells in CNS injuries via IL-10, TGF-β, etc. [19, 37]. Besides, Tregs-derived cytokines may interact directly with oligodendrocytes and facilitate myelin repair [23]. Although rarely distributed in CNS, Tregs infiltrate into CNS after injury such as ischemic stroke, intracerebral hemorrhage, spinal cord injury and TBI. Similarly, Tregs infiltrated to demyelinated lesion in our LPC model. In present study, we labeled microglia with Iba1, which is also expressed in macrophages. Previous study has proved that although blood-derived macrophages infiltrate into CNS early after LPC-demyelination in spinal cord, microglia dominate lesion by 7 days after demyelination (~ 85%). Also, they found perivascular and meningeal macrophages make up only a small percentage [38]. These evidences suggest that in our LPC-demyelination model, most of Iba1^+^ cells were microglia at 10 days post-demyelination. We observed that depletion of Tregs induced more peripheral immune cells infiltration and exacerbated in situ microgliosis, which are in line with previous studies showing Tregs limit innate and adaptive immune cells infiltration, excessive astrogliosis and microgliosis in diverse animal models [21, 39]. Emerging evidences have suggested diverse and complex interactions between microglia and peripheral immune cells in acute CNS injuries and neurodegenerative diseases [40]. After CNS injury, microglia are activated quickly and participate in peripheral immune cells recruitment. As the first immune cells to respond to damage and enter CNS, neutrophils can be engulfed by microglia. On the other side, depletion of neutrophils decreases microgliosis and improves cognitive function in AD model mice [41]. Monocyte-derived macrophages infiltrate into CNS 2–3 days post-injury and suppress microglial activation and phagocytosis [42]. However, it has also been found that infected macrophages can prime microglia and induce microglial activation of NLRP3 inflammasome [43]. Moreover, T and B lymphocytes deficiency inhibit microglia/macrophages activation in spinal cord injury [44]. In summary, peripheral immune cells interact delicately with microglia and influence microglial activation and function in CNS diseases, which can be regulated by Tregs in diverse pathological conditions. Previous studies have revealed Tregs alleviate neuroinflammation by inhibiting pro-inflammatory cytokines in vivo and in vitro [19, 45]. Consistently, our study showed remarkably increased expressions of pro-inflammatory cytokines including TNF-α, IL-6, IFN-γ and IL-1β after Tregs depletion in LPC-induced demyelination. We also observed a decrease in mature oligodendrocytes in lesion as well as lower protein expression of MBP, NF-H and NF-M after Tregs depletion, which may be attributed to the exacerbated inflammatory responses, since pro-inflammatory cytokines such as TNF-α, IL-6, IFN-γ and IL-1β may induce demyelination and inhibit remyelination in CNS [2, 46, 47].

Pyroptosis has been observed in multiple CNS diseases. Inhibition of pyroptosis components caspase-1, inflammasomes and gasdermin has been proved to confer protection in cerebral ischemia [11], AD [12], PD [48], developmental white matter injury [49] and EAE [10]. In line with their results, we found that microglial pyroptosis occurs in LPC-induced demyelination, and depletion of Tregs exacerbated microglial pyroptosis accompanied by aggravated myelin injury. As an inflammatory type of programmed cell death, whether pyroptosis can be regulated by Tregs has not been illustrated yet. Nonetheless, two recent studies have found drugs can regulate function of Tregs and pyroptosis simultaneously in lung injury [50, 51], and IL-10 neutralizing antibodies increased the expressions of pyroptosis-related proteins [50], suggesting the potential link between Tregs and pyroptosis. For the first time, our study substantiates that Tregs directly inhibit microglial pyroptosis in LPC-induced demyelination model via TLR4/MyD88/NF-κB pathway. Canonical inflammasome activation activates caspase-1 and triggers pyroptosis. Multiple inflammasomes are involved in CNS diseases. Previous studies have found microglia express multiple inflammasomes including NLRP3, NLRP1, NLRC4 and absent in melanoma 2 (AIM2), which mediate neuroinflammation in CNS diseases [4, 48, 52, 53]. Among these inflammasomes, NLRP3 was the first to be discovered in the brain and is mainly expressed by microglia [54]. NLRP3 can sense a series of stimuli from viral infection, lysosomal damage, metabolism dysfunction and extracellular ATP [55]. Similarly, we found caspase-1, GSDMD, NLRP3 and IL-1β mainly localize in microglia, suggesting microglia as the dominating cell type that undergo pyroptosis in our LPC-induced demyelination model. TLR4/MyD88/NF-κB pathway is widely involved in inflammatory responses and has also been proved to induce pyroptosis by facilitating the formation of NLRP3 inflammasome complex in CNS diseases [56, 57]. Consistently, we found that inhibiting TLR4 attenuated protein expression of NLRP3 and cleaved caspase-1 in LPC-demyelination model. Therefore, this study reveals that Tregs regulate pyroptosis via TLR4/MyD88/NF-κB pathway.

Pyroptosis aggravated myelin injury in various diseases including MS/EAE [10] and spinal cord injury [27]. In addition, pyroptosis components GSDMD and inflammasome directly induced EAE and white matter injury [49, 58], while inhibition of inflammasomes or caspase-1 both preserve myelin during injury [59–61]. Cells underwent pyroptosis release pro-inflammatory cytokines IL-1β and IL-18 at last. Our results of immunofluorescence confirmed increased IL-1β and Iba1 positive cells after LPC-induced demyelination, which was further increased after Tregs depletion. Emerging evidence increasingly indicates that IL-1β, which can be released by cells undergoing pyroptosis, impedes white matter repair and oligodendrocytes survival [30, 62]. In agreement with these findings, our results reveal caspase-1 inhibitor VX765 restored the myelin as well as cognitive function partially in Tregs-depleted mice, indicating that Tregs protect against myelin injury via regulating microglial pyroptosis in LPC-induced demyelination. Notably, VX765 significantly decreased the expression of caspase-1, cleaved caspase-1, GSDMD, cleaved GSDMD and IL-1β without affecting protein expression of NLRP3 in Tregs-depleted demyelinated mice. This discrepancy may be ascribed to caspase-1 activation as the downstream effector of inflammasomes activation. And our results are in line with a previous study who found VX765 had no influence on expressions of NLRP1, NLRP3, NLRP4 or AIM2 in TBI [63].

Besides regulating neuroinflammation and pyroptosis observed in our study, Tregs have exhibited pro-repair ability in multiple tissues including remyelination in CNS [64, 65]. A Previous study has found that CCN3 derived from Tregs directly promotes OPCs differentiation [23], while another study reveals that Tregs-derived osteopontin facilitates white matter repair by regulating microglia activity [22], and here in a new sight, we suggest that Tregs protect against myelin injury by inhibiting microglial pyroptosis in LPC-induced demyelination. However, the underlying mechanism how regulation of pyroptosis functions on myelin injury and repair is unclear and warrants further exploration.

Although depletion of Tregs by DT injection in DEREG mice has been widely used, recent studies have found Foxp3-negative Tregs with immunoregulatory capacity in multiple diseases, including CD4^+^ type 1 T regulatory (Tr1) cells, Th3 cells, CD8^+^ Tregs, et al. [66–68]. Depletion of these Foxp3-negative Tregs has not been performed in animal models yet. Further studies to explore novel methods for Tregs depletion are warranted to study functions of Tregs more accurately.

## Conclusions

In summary, we observed microglial pyroptosis in LPC-induced demyelination, and by abolishing Tregs and applying caspase-1 inhibitor VX765, we demonstrated that Tregs protect against myelin injury and cognitive function impairments via refraining microglial pyroptosis and excessive inflammatory responses for the first time. With VX765 treatment, we verified the indispensable role of pyroptosis in Tregs-conferred protection on myelin in our LPC-induced demyelination model. Furthermore, we applied RNA-sequencing and revealed Tregs regulate pyroptosis of microglia via TLR4/MyD88/NF-κB pathway. Taken together, we indicated a tight link between pyroptosis and Tregs which delicately regulates myelin injury and repair. Nonetheless, further studies to explore how microglial pyroptosis regulates myelin injury and repair are warranted.

## Supplementary Information

**Additional file 1: Table S1.** Primers used in qRT-PCR. **Figure S1.** DT successfully depletes Tregs in DEREG mice. **A **Representative flowcytometric images of CD4^+^Foxp3^+^ Tregs in blood and spleen from DEREG mice treated by PBS and DT. **B**, **C **Proportion of Tregs in CD4^+^ T cells in blood (**B**) and spleen (**C**) of DEREG mice treated by PBS and DT. *N*=4/group, two-tailed Student’s t-test was used, ***p<0.001. **Figure S2.** Oligodendrocytes and astrocytes rarely undergo pyroptosis in LPC-induced demyelination. **A**, **B** Representative immunofluorescent double-labeling of Olig2 and GSDMD (**A**), GFAP and GSDMD (**B**) in lesion respectively, enlarged images of single channel were shown. Scale bar=50 μm. **Figure S3.** RNA-sequencing reveals that depletion of Tregs significantly influences immune system and inflammatory response in LPC-induced demyelination. **A**, **B** Volcano plot of DEGs between sham and PBS-LPC group (**A**) as well as LPC group treated by PBS and DT (**B**). **C** Heat map of DEGs among groups. **D** Top 20 pathways enriched by GO biological processes analysis of DEGs common in sham VS PBS-LPC and PBS-LPC VS DT-LPC. For RNA-sequencing analysis, n=5 replicates for each group. **Figure S4.** The top ten key genes of KDA in RNA-seq were verified by qPCR. N=4-5/group, one-way ANOVA with Bonferroni’s test. *P<0.05, **p<0.01, ***p<0.001, ****p<0.0001.

**Additional file 2:** Raw images of western blot. The original western blots for Figure 2F. The original western blots for Figure 3B. The original western blots for Figure 4E.The original western blots for Figure 5E.The original western blots for Figure 7F.

## Data Availability

The datasets used and/or analyzed during the current study are available from the corresponding author on reasonable request.

## Abbreviations

CNS: Central nervous system
Tregs: Regulatory T cells
DTR: Diphtheria toxin receptor
DT: Diphtheria toxin
LPC: Lysophosphatidylcholine
LFB: Luxol fast blue
qRT-PCR: Quantitative real-time PCR
MyD88: Myeloid differentiation marker 88
IL-1β: Interleukin-1β
TNF-α: Tumor necrosis factor-α
IFN-γ: Interferon-γ
GSDMD: Gasdermin D
IL-18: Interleukin-18
MS: Multiple sclerosis
AD: Alzheimer’s disease
TBI: Traumatic brain injury
NLRP3: Nod-like receptor family, pyrin domain containing 3
EAE: Experimental autoimmune encephalomyelitis
PD: Parkinson disease
IL-10: Interleukin-10
TGF-β: Transforming growth factor-β
IL-35: Interleukin-35
CC: Corpus callosum
Dpi: Days post-injection
PFA: Paraformaldehyde
Iba1: Ionized calcium-binding adapter molecule 1
DAPI: 4,6-Diamidino-2-phenylindole
SDS-PAGE: Sodium dodecyl sulfate-polyacrylamide gel electrophoresis
NLRC4: Nod-like receptor family, CARD domain containing 4
OD: Optical densities
DI: Discrimination index
HISAT: Hierarchical Indexing for Spliced Alignment of Transcripts
DEGs: Differentially expressed genes
PPI: Protein–protein interaction
GSEA: Gene set enrichment analysis
KDA: Key driver analysis
SD: Standard deviation
ANOVA: Analysis of variance
BBB: Blood–brain barrier
OPCs: Oligodendrocyte progenitor cells
ALS: Amyotrophic lateral sclerosis
IL-6: Interleukin-6
NLRP1: Nod-like receptor family, pyrin domain containing 1
TLRs: Toll-like receptors
PAMPs: Pathogen-associated molecular patterns
DAMPs: Damage-associated molecular patterns
AIM2: Absent in melanoma 2

## References

[1] Stadelmann C, Timmler S, Barrantes-Freer A, Simons M (2019). Myelin in the central nervous system: structure, function, and pathology. Physiol Rev.

[2] Plemel JR, Liu WQ, Yong VW (2017). Remyelination therapies: a new direction and challenge in multiple sclerosis. Nat Rev Drug Discov.

[3] Ljubisavljevic S (2016). Oxidative Stress and Neurobiology of Demyelination. Mol Neurobiol.

[4] Freeman L, Guo H, David CN, Brickey WJ, Jha S, Ting JP (2017). NLR members NLRC4 and NLRP3 mediate sterile inflammasome activation in microglia and astrocytes. J Exp Med.

[5] Rutkowska A, Sailer AW, Dev KK (2017). EBI2 receptor regulates myelin development and inhibits LPC-induced demyelination. J Neuroinflammation.

[6] Shi J, Gao W, Shao F (2017). Pyroptosis: gasdermin-mediated programmed necrotic cell death. Trends Biochem Sci.

[7] Kovacs SB, Miao EA (2017). Gasdermins: effectors of pyroptosis. Trends Cell Biol.

[8] Amarante-Mendes GP, Adjemian S, Branco LM, Zanetti LC, Weinlich R, Bortoluci KR (2018). Pattern recognition receptors and the host cell death molecular machinery. Front Immunol.

[9] McKenzie BA, Dixit VM, Power C (2020). Fiery cell death: pyroptosis in the central nervous system. Trends Neurosci.

[10] McKenzie BA, Mamik MK, Saito LB, Boghozian R, Monaco MC, Major EO, Lu JQ, Branton WG, Power C (2018). Caspase-1 inhibition prevents glial inflammasome activation and pyroptosis in models of multiple sclerosis. Proc Natl Acad Sci U S A.

[11] Li J, Hao JH, Yao D, Li R, Li XF, Yu ZY, Luo X, Liu XH, Wang MH, Wang W (2020). Caspase-1 inhibition prevents neuronal death by targeting the canonical inflammasome pathway of pyroptosis in a murine model of cerebral ischemia. CNS Neurosci Ther.

[12] Flores J, Noel A, Foveau B, Lynham J, Lecrux C, LeBlanc AC (2018). Caspase-1 inhibition alleviates cognitive impairment and neuropathology in an Alzheimer's disease mouse model. Nat Commun.

[13] Irrera N, Pizzino G, Calò M, Pallio G, Mannino F, Famà F, Arcoraci V, Fodale V, David A, Francesca C (2017). Lack of the Nlrp3 inflammasome improves mice recovery following traumatic brain injury. Front Pharmacol.

[14] Cui J, Zhao S, Li Y, Zhang D, Wang B, Xie J, Wang J (2021). Regulated cell death: discovery, features and implications for neurodegenerative diseases. Cell Commun Signal.

[15] Peng Y, Chen J, Dai Y, Jiang Y, Qiu W, Gu Y, Wang H (2019). NLRP3 level in cerebrospinal fluid of patients with neuromyelitis optica spectrum disorders: Increased levels and association with disease severity. Mult Scler Relat Disord.

[16] Sakaguchi S, Yamaguchi T, Nomura T, Ono M (2008). Regulatory T cells and immune tolerance. Cell.

[17] Vignali DA, Collison LW, Workman CJ (2008). How regulatory T cells work. Nat Rev Immunol.

[18] Raffin C, Vo LT, Bluestone JA (2020). Treg cell-based therapies: challenges and perspectives. Nat Rev Immunol.

[19] Ito M, Komai K, Mise-Omata S, Iizuka-Koga M, Noguchi Y, Kondo T, Sakai R, Matsuo K, Nakayama T, Yoshie O (2019). Brain regulatory T cells suppress astrogliosis and potentiate neurological recovery. Nature.

[20] Zhou K, Zhong Q, Wang YC, Xiong XY, Meng ZY, Zhao T, Zhu WY, Liao MF, Wu LR, Yang YR (2017). Regulatory T cells ameliorate intracerebral hemorrhage-induced inflammatory injury by modulating microglia/macrophage polarization through the IL-10/GSK3beta/PTEN axis. J Cereb Blood Flow Metab.

[21] Kramer TJ, Hack N, Bruhl TJ, Menzel L, Hummel R, Griemert EV, Klein M, Thal SC, Bopp T, Schafer MKE (2019). Depletion of regulatory T cells increases T cell brain infiltration, reactive astrogliosis, and interferon-gamma gene expression in acute experimental traumatic brain injury. J Neuroinflammation.

[22] Shi L, Sun Z, Su W, Xu F, Xie D, Zhang Q, Dai X, Iyer K, Hitchens TK, Foley LM (2021). Treg cell-derived osteopontin promotes microglia-mediated white matter repair after ischemic stroke. Immunity.

[23] Dombrowski Y, O'Hagan T, Dittmer M, Penalva R, Mayoral SR, Bankhead P, Fleville S, Eleftheriadis G, Zhao C, Naughton M (2017). Regulatory T cells promote myelin regeneration in the central nervous system. Nat Neurosci.

[24] Luo Q, Ding L, Zhang N, Jiang Z, Gao C, Xue L, Peng B, Wang G (2018). A stable and easily reproducible model of focal white matter demyelination. J Neurosci Methods.

[25] Kamiya M, Mizoguchi F, Kawahata K, Wang D, Nishibori M, Day J, Louis C, Wicks IP, Kohsaka H, Yasuda S (2022). Targeting necroptosis in muscle fibers ameliorates inflammatory myopathies. Nat Commun.

[26] Mo Y-Q, Nakamura H, Tanaka T, Odani T, Perez P, Ji Y, French BN, Pranzatelli TJF, Michael DG, Yin H (2022). Lysosomal exocytosis of HSP70 stimulates monocytic BMP6 expression in Sjögren’s syndrome. J Clin Invest.

[27] Li X, Yu Z, Zong W, Chen P, Li J, Wang M, Ding F, Xie M, Wang W, Luo X (2020). Deficiency of the microglial Hv1 proton channel attenuates neuronal pyroptosis and inhibits inflammatory reaction after spinal cord injury. J Neuroinflammation.

[28] Vorhees CV, Williams MT (2006). Morris water maze: procedures for assessing spatial and related forms of learning and memory. Nat Protoc.

[29] Chen M, Yang LL, Hu ZW, Qin C, Zhou LQ, Duan YL, Bosco DB, Wu LJ, Zhan KB, Xu SB, Tian DS (2020). Deficiency of microglial Hv1 channel is associated with activation of autophagic pathway and ROS production in LPC-induced demyelination mouse model. J Neuroinflammation.

[30] Zhou Y, Zhang J, Wang L, Chen Y, Wan Y, He Y, Jiang L, Ma J, Liao R, Zhang X (2017). Interleukin-1beta impedes oligodendrocyte progenitor cell recruitment and white matter repair following chronic cerebral hypoperfusion. Brain Behav Immun.

[31] Huang Y, Xu W, Zhou R (2021). NLRP3 inflammasome activation and cell death. Cell Mol Immunol.

[32] Xue Y, Enosi Tuipulotu D, Tan WH, Kay C, Man SM (2019). Emerging activators and regulators of inflammasomes and pyroptosis. Trends Immunol.

[33] Qin C, Liu Q, Hu ZW, Zhou LQ, Shang K, Bosco DB, Wu LJ, Tian DS, Wang W (2018). Microglial TLR4-dependent autophagy induces ischemic white matter damage via STAT1/6 pathway. Theranostics.

[34] Papageorgiou IE, Lewen A, Galow LV, Cesetti T, Scheffel J, Regen T, Hanisch UK, Kann O (2016). TLR4-activated microglia require IFN-γ to induce severe neuronal dysfunction and death in situ. Proc Natl Acad Sci U S A.

[35] O'Neill LA, Bowie AG (2007). The family of five: TIR-domain-containing adaptors in Toll-like receptor signalling. Nat Rev Immunol.

[36] Lin C, Wang H, Zhang M, Mustafa S, Wang Y, Li H, Yin H, Hutchinson MR, Wang X (2021). TLR4 biased small molecule modulators. Pharmacol Ther.

[37] Liesz A, Hu X, Kleinschnitz C, Offner H (2015). Functional role of regulatory lymphocytes in stroke: facts and controversies. Stroke.

[38] Plemel JR, Stratton JA, Michaels NJ, Rawji KS, Zhang E, Sinha S, Baaklini CS, Dong Y, Ho M, Thorburn K (2020). Microglia response following acute demyelination is heterogeneous and limits infiltrating macrophage dispersion. Sci Adv.

[39] Liesz A, Suri-Payer E, Veltkamp C, Doerr H, Sommer C, Rivest S, Giese T, Veltkamp R (2009). Regulatory T cells are key cerebroprotective immunomodulators in acute experimental stroke. Nat Med.

[40] Greenhalgh AD, David S, Bennett FC (2020). Immune cell regulation of glia during CNS injury and disease. Nat Rev Neurosci.

[41] Zenaro E, Pietronigro E, Della Bianca V, Piacentino G, Marongiu L, Budui S, Turano E, Rossi B, Angiari S, Dusi S (2015). Neutrophils promote Alzheimer's disease-like pathology and cognitive decline via LFA-1 integrin. Nat Med.

[42] Greenhalgh AD, Zarruk JG, Healy LM, Baskar Jesudasan SJ, Jhelum P, Salmon CK, Formanek A, Russo MV, Antel JP, McGavern DB (2018). Peripherally derived macrophages modulate microglial function to reduce inflammation after CNS injury. PLoS Biol.

[43] Lee HM, Kang J, Lee SJ, Jo EK (2013). Microglial activation of the NLRP3 inflammasome by the priming signals derived from macrophages infected with mycobacteria. Glia.

[44] Wu B, Matic D, Djogo N, Szpotowicz E, Schachner M, Jakovcevski I (2012). Improved regeneration after spinal cord injury in mice lacking functional T- and B-lymphocytes. Exp Neurol.

[45] Xie L, Choudhury GR, Winters A, Yang SH, Jin K (2015). Cerebral regulatory T cells restrain microglia/macrophage-mediated inflammatory responses via IL-10. Eur J Immunol.

[46] Correale J, Gaitan MI, Ysrraelit MC, Fiol MP (2017). Progressive multiple sclerosis: from pathogenic mechanisms to treatment. Brain.

[47] Ramesh G, MacLean AG, Philipp MT (2013). Cytokines and chemokines at the crossroads of neuroinflammation, neurodegeneration, and neuropathic pain. Mediators Inflamm.

[48] Gordon R, Albornoz EA, Christie DC, Langley MR, Kumar V, Mantovani S, Robertson AAB, Butler MS, Rowe DB, O'Neill LA (2018). Inflammasome inhibition prevents alpha-synuclein pathology and dopaminergic neurodegeneration in mice. Sci Transl Med.

[49] Holloway RK, Ireland G, Sullivan G, Becher JC, Smith C, Boardman JP, Gressens P, Miron VE (2021). Microglial inflammasome activation drives developmental white matter injury. Glia.

[50] Zhang ZT, Zhang DY, Xie K, Wang CJ, Xu F (2021). Luteolin activates Tregs to promote IL-10 expression and alleviating caspase-11-dependent pyroptosis in sepsis-induced lung injury. Int Immunopharmacol.

[51] Xie K, Chen YQ, Chai YS, Lin SH, Wang CJ, Xu F (2021). HMGB1 suppress the expression of IL-35 by regulating Naïve CD4+ T cell differentiation and aggravating Caspase-11-dependent pyroptosis in acute lung injury. Int Immunopharmacol.

[52] Poh L, Kang SW, Baik SH, Ng GYQ, She DT, Balaganapathy P, Dheen ST, Magnus T, Gelderblom M, Sobey CG (2019). Evidence that NLRC4 inflammasome mediates apoptotic and pyroptotic microglial death following ischemic stroke. Brain Behav Immun.

[53] Kim H, Seo JS, Lee SY, Ha KT, Choi BT, Shin YI, Ju Yun Y, Shin HK (2020). AIM2 inflammasome contributes to brain injury and chronic post-stroke cognitive impairment in mice. Brain Behav Immun.

[54] Heneka MT, McManus RM, Latz E (2018). Inflammasome signalling in brain function and neurodegenerative disease. Nat Rev Neurosci.

[55] Mangan MSJ, Olhava EJ, Roush WR, Seidel HM, Glick GD, Latz E (2018). Targeting the NLRP3 inflammasome in inflammatory diseases. Nat Rev Drug Discov.

[56] Yang J, Wise L, Fukuchi K-I (2020). TLR4 cross-talk with NLRP3 inflammasome and complement signaling pathways in Alzheimer's disease. Front Immunol.

[57] Luo L, Liu M, Fan Y, Zhang J, Liu L, Li Y, Zhang Q, Xie H, Jiang C, Wu J (2022). Intermittent theta-burst stimulation improves motor function by inhibiting neuronal pyroptosis and regulating microglial polarization via TLR4/NFkappaB/NLRP3 signaling pathway in cerebral ischemic mice. J Neuroinflammation.

[58] Li S, Wu Y, Yang D, Wu C, Ma C, Liu X, Moynagh PN, Wang B, Hu G, Yang S (2019). Gasdermin D in peripheral myeloid cells drives neuroinflammation in experimental autoimmune encephalomyelitis. J Exp Med.

[59] Poh L, Fann DY, Wong P, Lim HM, Foo SL, Kang SW, Rajeev V, Selvaraji S, Iyer VR, Parathy N (2020). AIM2 inflammasome mediates hallmark neuropathological alterations and cognitive impairment in a mouse model of vascular dementia. Mol Psychiatry.

[60] Amo-Aparicio J, Garcia-Garcia J, Puigdomenech M, Francos-Quijorna I, Skouras DB, Dinarello CA, Lopez-Vales R (2022). Inhibition of the NLRP3 inflammasome by OLT1177 induces functional protection and myelin preservation after spinal cord injury. Exp Neurol.

[61] Saito LB, Fernandes JP, Smith MJ, Doan MAL, Branton WG, Schmitt LM, Wuest M, Monaco MC, Major EO, Wuest F, Power C (2021). Intranasal anti-caspase-1 therapy preserves myelin and glucose metabolism in a model of progressive multiple sclerosis. Glia.

[62] Kelly SB, Stojanovska V, Zahra VA, Moxham A, Miller SL, Moss TJM, Hooper SB, Nold MF, Nold-Petry CA, Dean JM (2021). Interleukin-1 blockade attenuates white matter inflammation and oligodendrocyte loss after progressive systemic lipopolysaccharide exposure in near-term fetal sheep. J Neuroinflammation.

[63] Sun Z, Nyanzu M, Yang S, Zhu X, Wang K, Ru J, Yu E, Zhang H, Wang Z, Shen J (2020). VX765 attenuates pyroptosis and HMGB1/TLR4/NF-kappaB pathways to improve functional outcomes in TBI mice. Oxid Med Cell Longev.

[64] de la Vega GN, Dittmer M, Dombrowski Y, Fitzgerald DC (2019). Regenerating CNS myelin: emerging roles of regulatory T cells and CCN proteins. Neurochem Int.

[65] Li J, Tan J, Martino MM, Lui KO (2018). Regulatory T-cells: potential regulator of tissue repair and regeneration. Front Immunol.

[66] Song Y, Wang N, Chen L, Fang L (2021). Tr1 cells as a key regulator for maintaining immune homeostasis in transplantation. Front Immunol.

[67] Gagliani N, Magnani CF, Huber S, Gianolini ME, Pala M, Licona-Limon P, Guo B, Herbert DR, Bulfone A, Trentini F (2013). Coexpression of CD49b and LAG-3 identifies human and mouse T regulatory type 1 cells. Nat Med.

[68] Krop J, Heidt S, Claas FHJ, Eikmans M (2020). Regulatory T cells in pregnancy: it is not all about FoxP3. Front Immunol.

