# Supplementary material for: Regulatory T cells alleviate myelin loss and cognitive dysfunction by regulating neuroinflammation and microglial pyroptosis via TLR4/MyD88/NF-κB pathway in LPC-induced demyelination
Source: J Neuroinflammation. 2023 Feb 18;20:41. doi: 10.1186/s12974-023-02721-0 (PMC9938996; doi:10.1186/s12974-023-02721-0)
Supplement: Supplementary file 1 — Additional file 1: Table S1. Primers used in qRT-PCR. Figure S1. DT successfully depletes Tregs in DEREG mice. A Representative flowcytometric images of CD4+Foxp3+ Tregs in blood and spleen from DEREG mice treated by PBS and DT. B, C Proportion of Tregs in CD4+ T cells in blood (B) and spleen (C) of DEREG mice treated by PBS and DT. N=4/group, two-tailed Student’s t-test was used, ***p<0.001. Figure S2. Oligodendrocytes and astrocytes rarely undergo pyroptosis in LPC-induced demyelination. A, B Representative immunofluorescent double-labeling of Olig2 and GSDMD (A), GFAP and GSDMD (B) in lesion respectively, enlarged images of single channel were shown. Scale bar=50 μm. Figure S3. RNA-sequencing reveals that depletion of Tregs significantly influences immune system and inflammatory response in LPC-induced demyelination. A, B Volcano plot of DEGs between sham and PBS-LPC group (A) as well as LPC group treated by PBS and DT (B). C Heat map of DEGs among groups. D Top 20 pathways enriched by GO biological processes analysis of DEGs common in sham VS PBS-LPC and PBS-LPC VS DT-LPC. For RNA-sequencing analysis, n=5 replicates for each group. Figure S4. The top ten key genes of KDA in RNA-seq were verified by qPCR. N=4-5/group, one-way ANOVA with Bonferroni’s test. *P<0.05, **p<0.01, ***p<0.001, ****p<0.0001. [file 12974_2023_2721_MOESM1_ESM.docx]

Additional file 1: Table S1: Primers used in qRT-PCR.

|  | Forward primers (5’-3’) | Reverse primers (5’-3’) |
| --- | --- | --- |
| *Tnfa* | ATGTCTCAGCCTCTTCTCATTC | GCTTGTCACTCGAATTTTGAGA |
| *Il6* | CTCCCAACAGACCTGTCTATAC | CCATTGCACAACTCTTTTCTCA |
| *Ifnr* | CTTGAAAGACAATCAGGCCATC | CTTGGCAATACTCATGAATGCA |
| *Il1b* | TCGCAGCAGCACATCAACAAGAG | AGGTCCACGGGAAAGACACAGG |
| *Tgfb* | CCAGATCCTGTCCAAACTAAGG | CTCTTTAGCATAGTAGTCCGCT |
| *Il4* | TACCAGGAGCCATATCCACGGATG | TGTGGTGTTCTTCGTTGCTGTGAG |
| *Aim2* | GTCACCAGTTCCTCAGTTGTG | CACCTCCATTGTCCCTGTTTTAT |
| *Nlrp3* | ATTACCCGCCCGAGAAAGG | TCGCAGCAAAGATCCACACAG |
| *Nlrc4* | ATCGTCATCACCGTGTGGAG | GCCAGACTCGCCTTCAATCA |
| *Nlrp1* | CCACTGAGCTACTATGCAGTACA | ACAACATCTTCACACCACCATC |
| *Irf9* | GCCGAGTGGTGGGTAAGAC | GCAAAGGCGCTGAACAAAGAG |
| *Casp4* | ACAAACACCCTGACAAACCAC | CACTGCGTTCAGCATTGTTAAA |
| *Myd88* | AGGACAAACGCCGGAACTTTT | GCCGATAGTCTGTCTGTTCTAGT |
| *Ifi204* | ATGCTTCATGCTACAGTGGCTACAG | TCATTGATCTCCAGGATGCCTTTGC |
| *Stat1* | TCACAGTGGTTCGAGCTTCAG | GCAAACGAGACATCATAGGCA |
| *Stat2* | TCCTGCCAATGGACGTTCG | GTCCCACTGGTTCAGTTGGT |
| *Irf7* | GAGACTGGCTATTGGGGGAG | GACCGAAATGCTTCCAGGG |
| *Ikbke* | ACCACTAACTACCTGTGGCAT | CCTCCCCGGATTTCTTGTTTC |
| *Gbp2* | CTGCACTATGTGACGGAGCTA | GAGTCCACACAAAGGTTGGAAA |


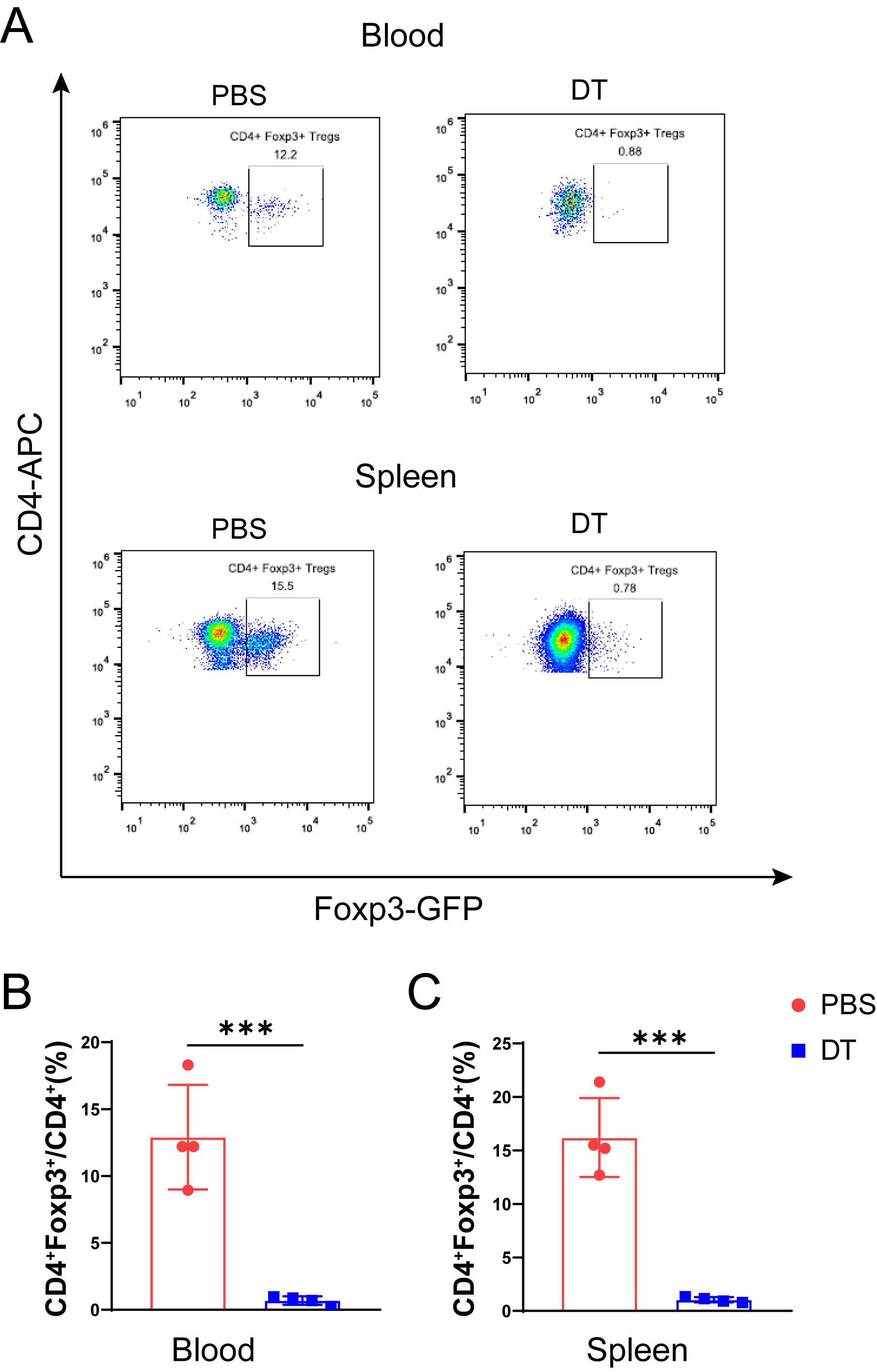


**Additional file 1:** **Figure S1:** DT successfully depletes Tregs in DEREG mice. **A** Representative flowcytometric images of CD4^+^Foxp3^+^ Tregs in blood and spleen from DEREG mice treated by PBS and DT. **B, C** Proportion of Tregs in CD4^+^ T cells in blood (B) and spleen (C) of DEREG mice treated by PBS and DT. N=4/group, two-tailed student’s t-test was used, ***p<0.001.


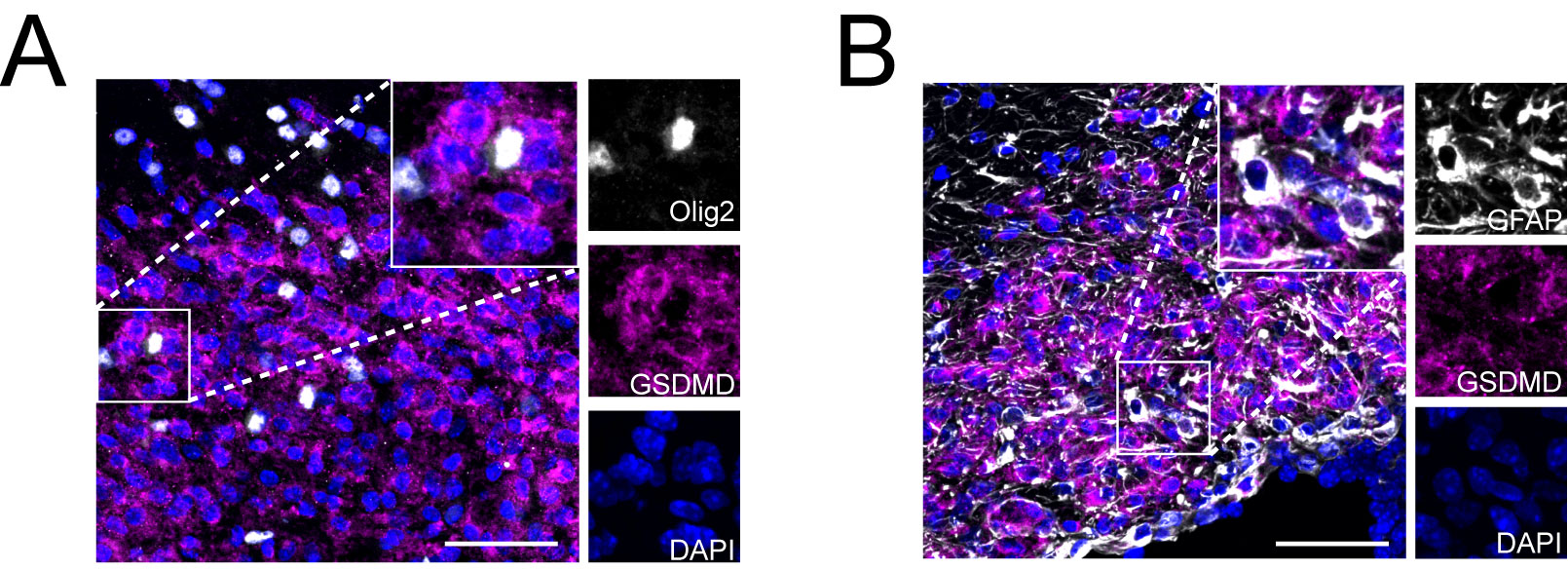


**Additional file 1:** **Figure S2:** Oligodendrocytes and astrocytes rarely undergo pyroptosis in LPC-induced demyelination. **A, B** Representative immunofluorescent double-labelling of Olig2 and GSDMD (A), GFAP and GSDMD (B) in lesion respectively, enlarged images of single channel were shown. Scale bar=50 μm.


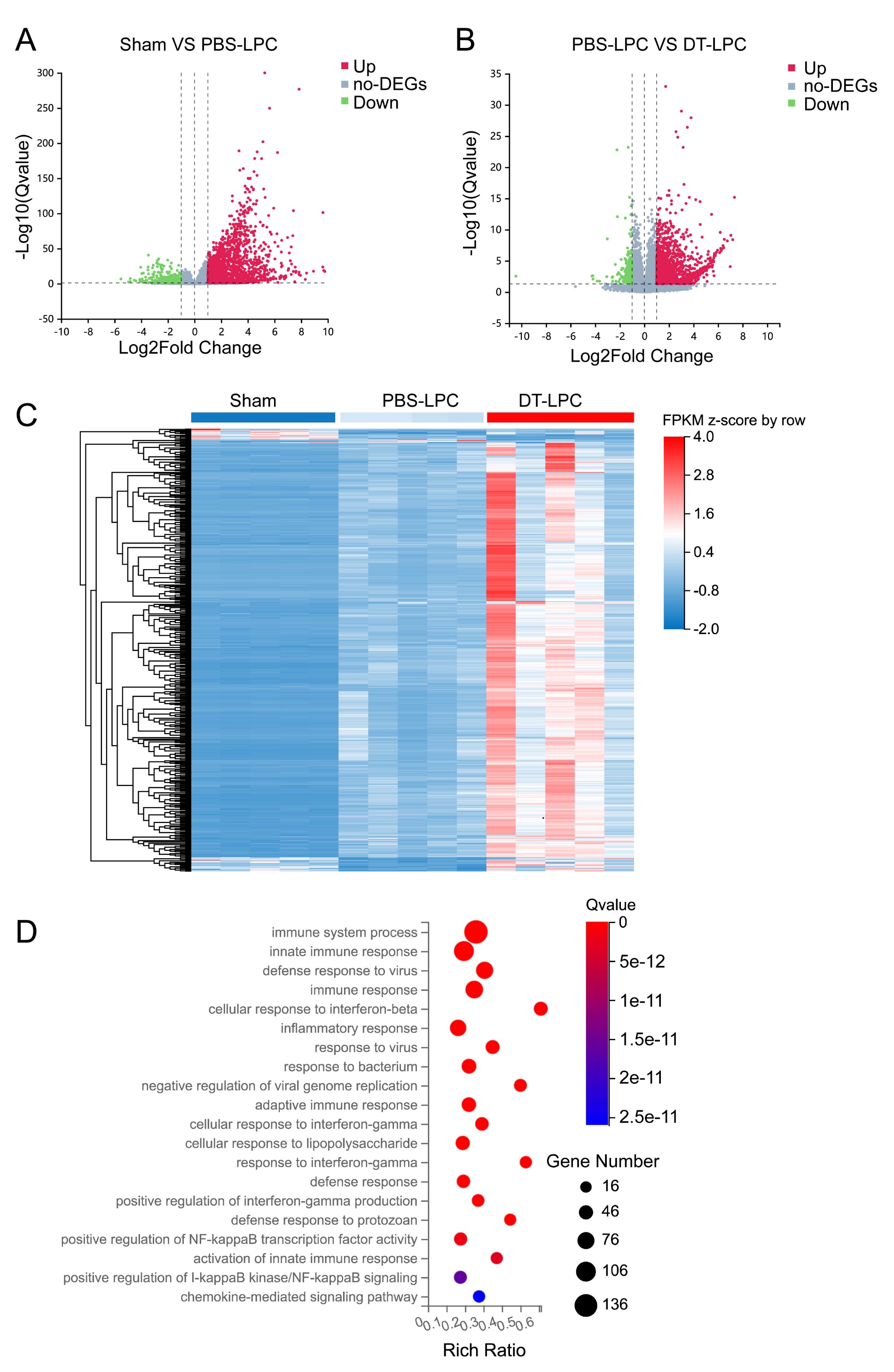


**Additional file 1:** **Figure S3:** RNA-sequencing reveals that depletion of Tregs significantly influences immune system and inflammatory response in LPC-induced demyelination. **A, B** Volcano plot of DEGs between sham and PBS-LPC group (A) as well as LPC group treated by PBS and DT (B). **C** Heat map of DEGs among groups. **D** Top 20 pathways enriched by GO biological processes analysis of DEGs common in sham VS PBS-LPC and PBS-LPC VS DT-LPC. For RNA-sequencing analysis, n=5 replicates for each group.


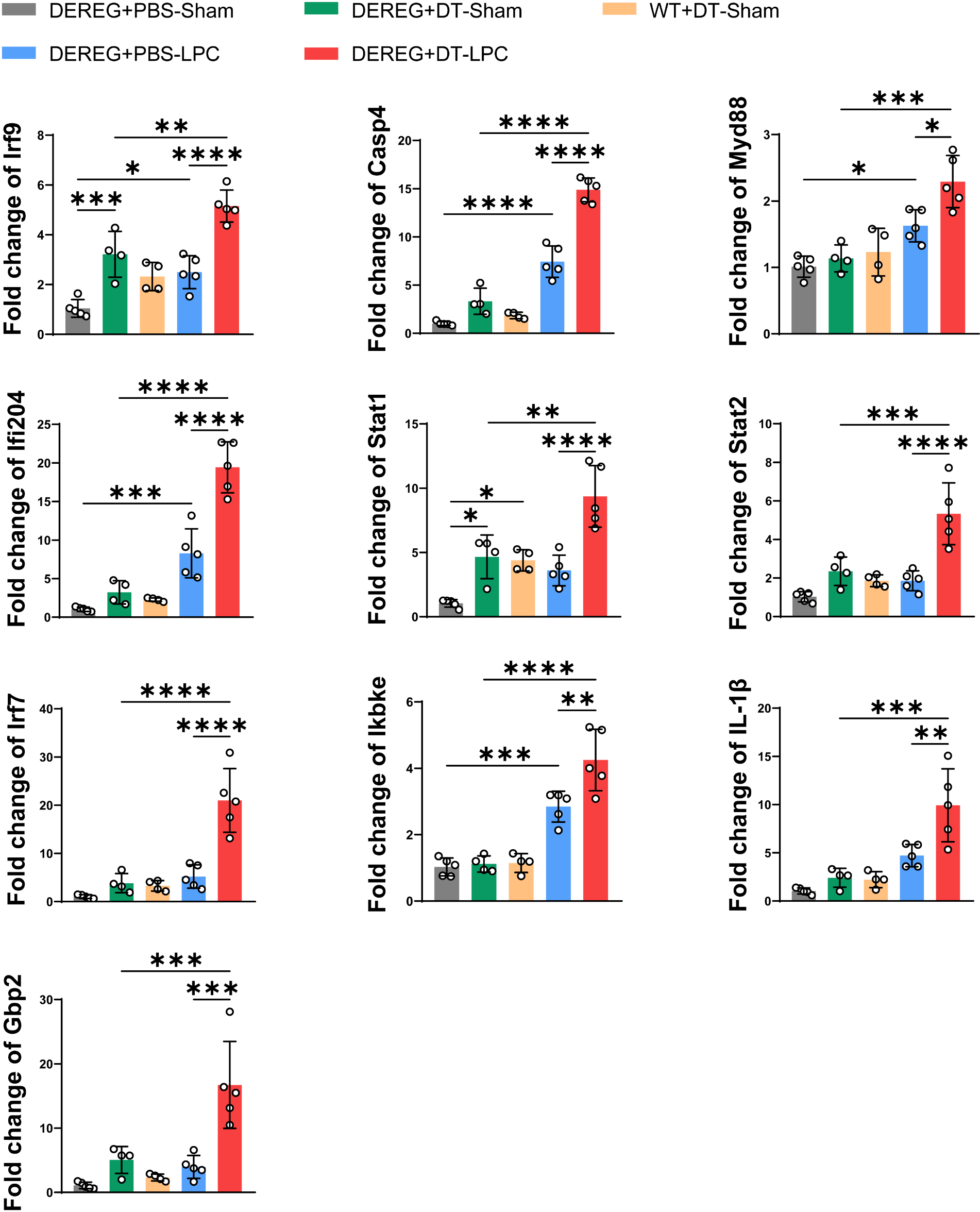


**Additional file 1:** **Figure S4:** The top ten key genes of KDA in RNA-seq were verified by qPCR. N=4-5/group, one-way ANOVA with Bonferroni’s test. *P<0.05, **p<0.01, ***p<0.001, ****p<0.0001.
