# Supplementary material for: Regulatory T cells alleviate myelin loss and cognitive dysfunction by regulating neuroinflammation and microglial pyroptosis via TLR4/MyD88/NF-κB pathway in LPC-induced demyelination
Source: J Neuroinflammation. 2023 Feb 18;20:41. doi: 10.1186/s12974-023-02721-0 (PMC9938996; doi:10.1186/s12974-023-02721-0)
Supplement: Supplementary file 2 — Additional file 2: Raw images of western blot. The original western blots for Figure 2F. The original western blots for Figure 3B. The original western blots for Figure 4E.The original western blots for Figure 5E.The original western blots for Figure 7F. [file 12974_2023_2721_MOESM2_ESM.docx]

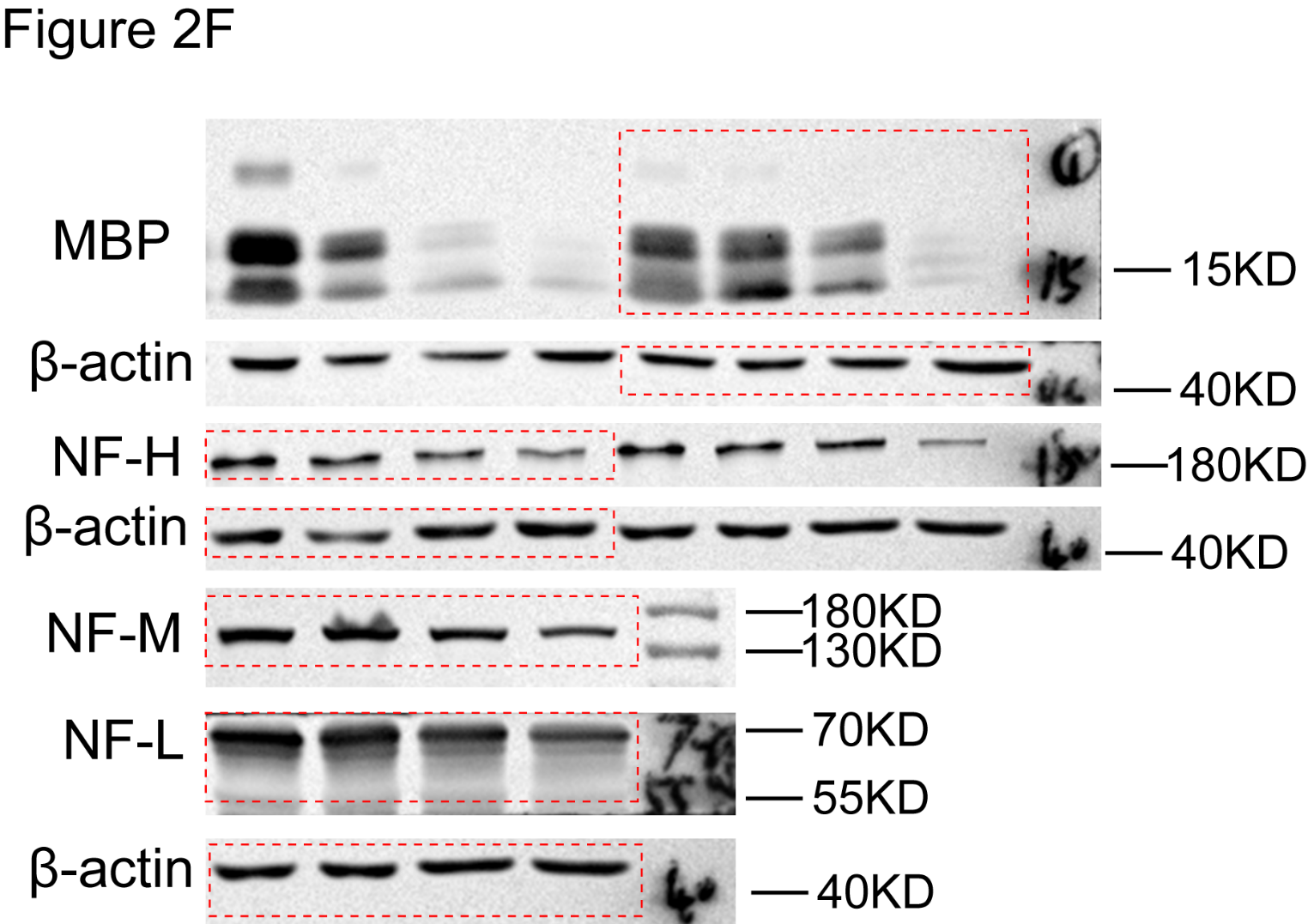


The original western blots for Figure. 2F.


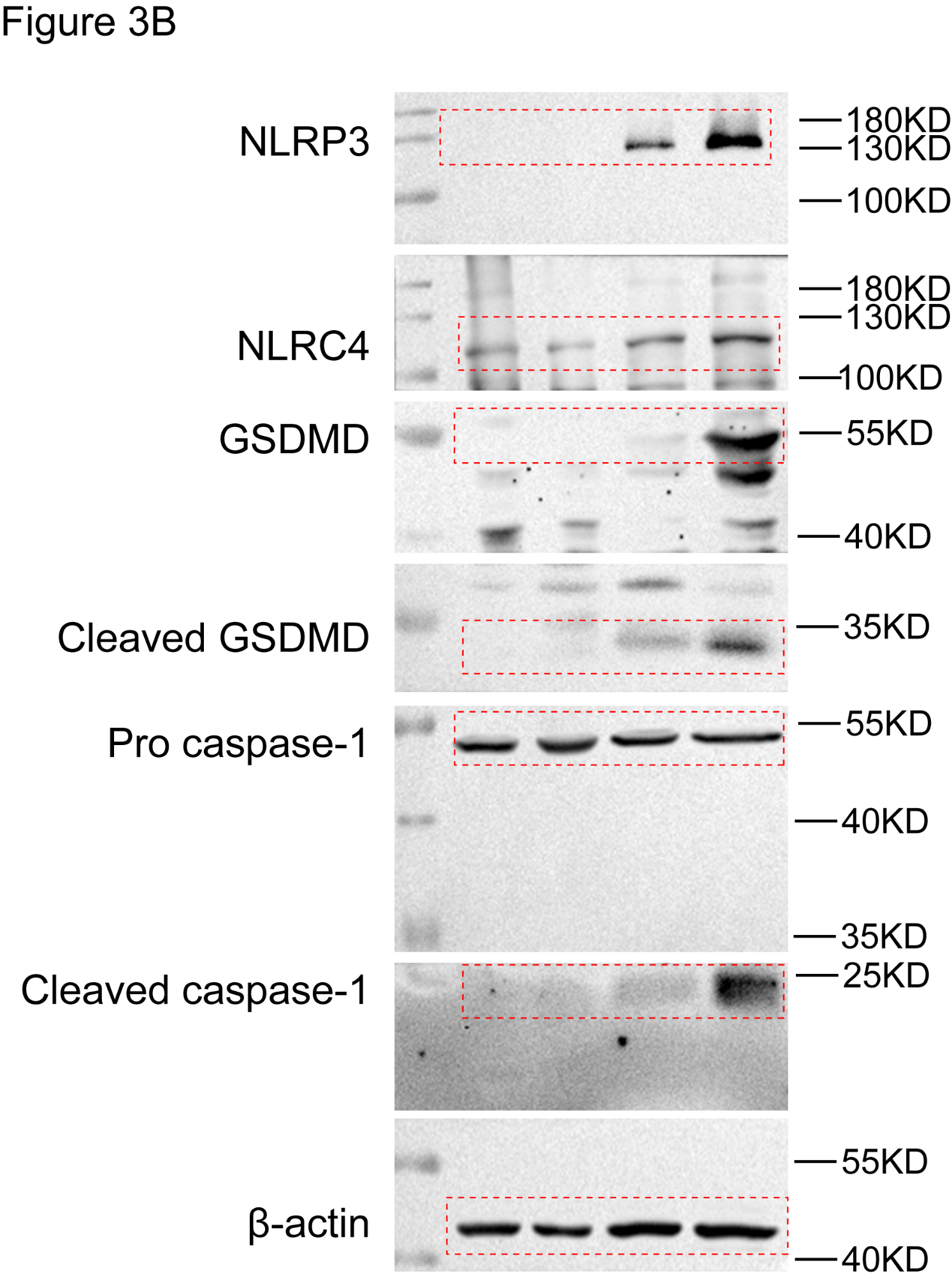


The original western blots for Figure. 3B.


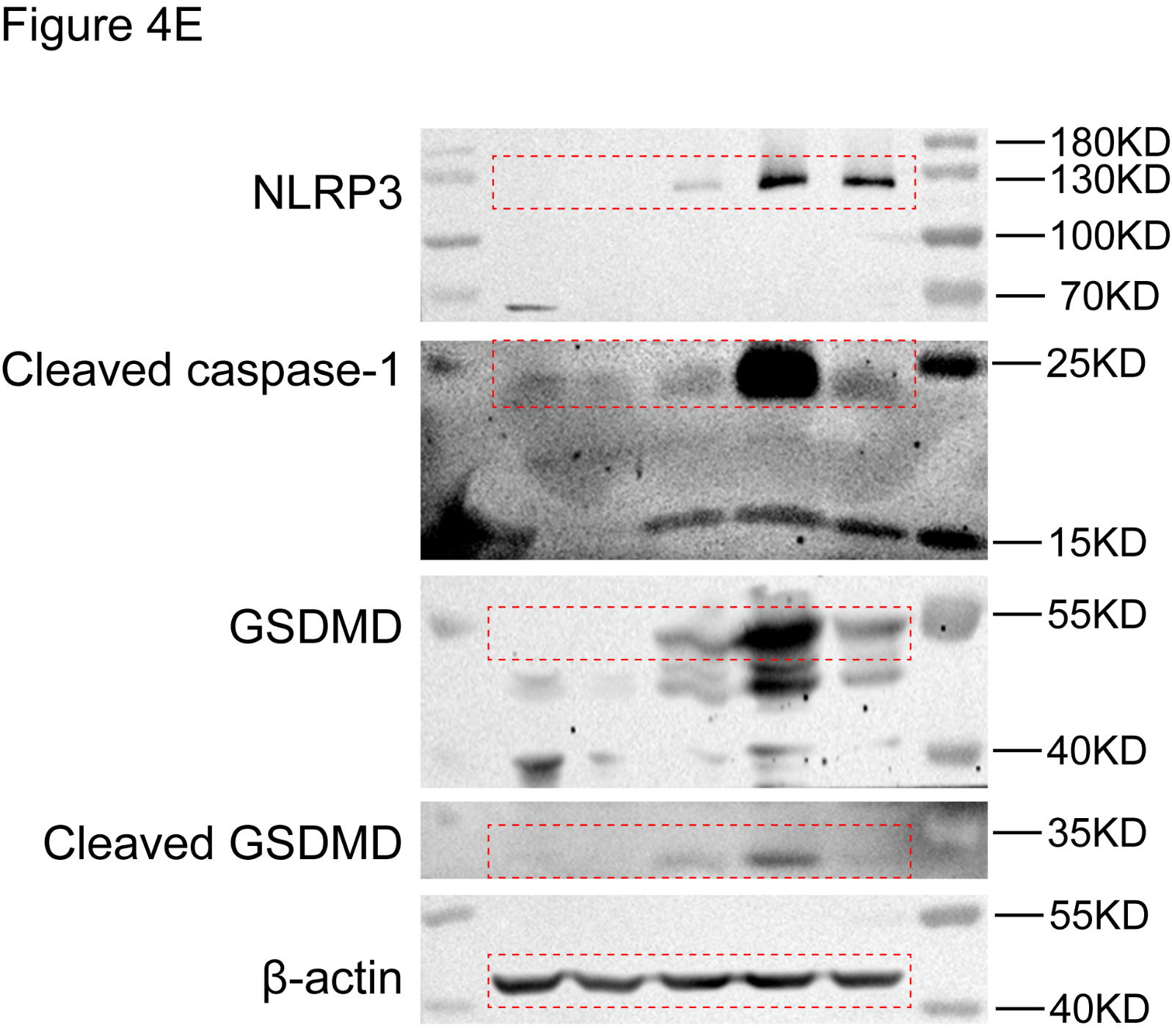


The original western blots for Figure. 4E.


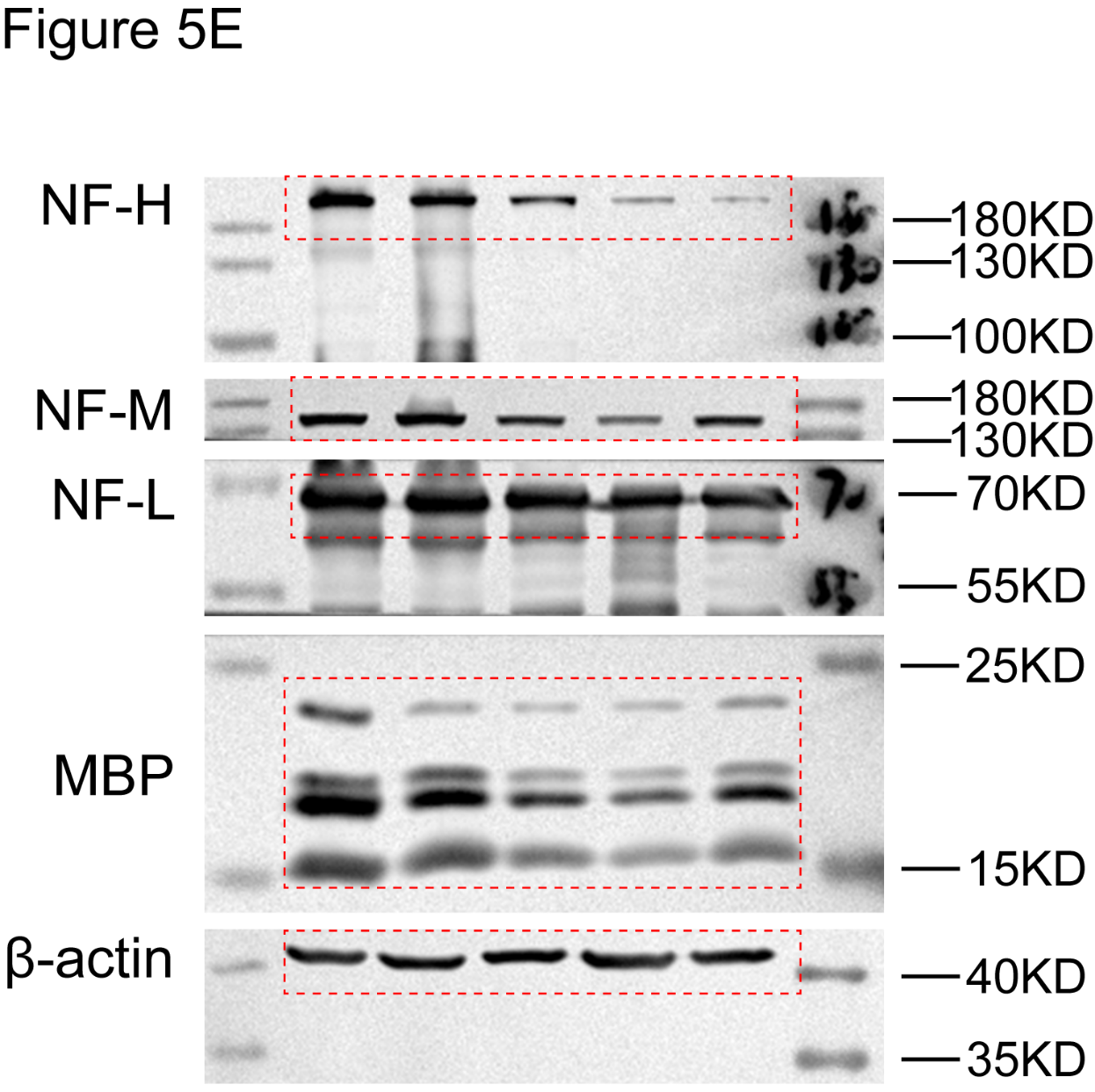


The original western blots for Figure. 5E.


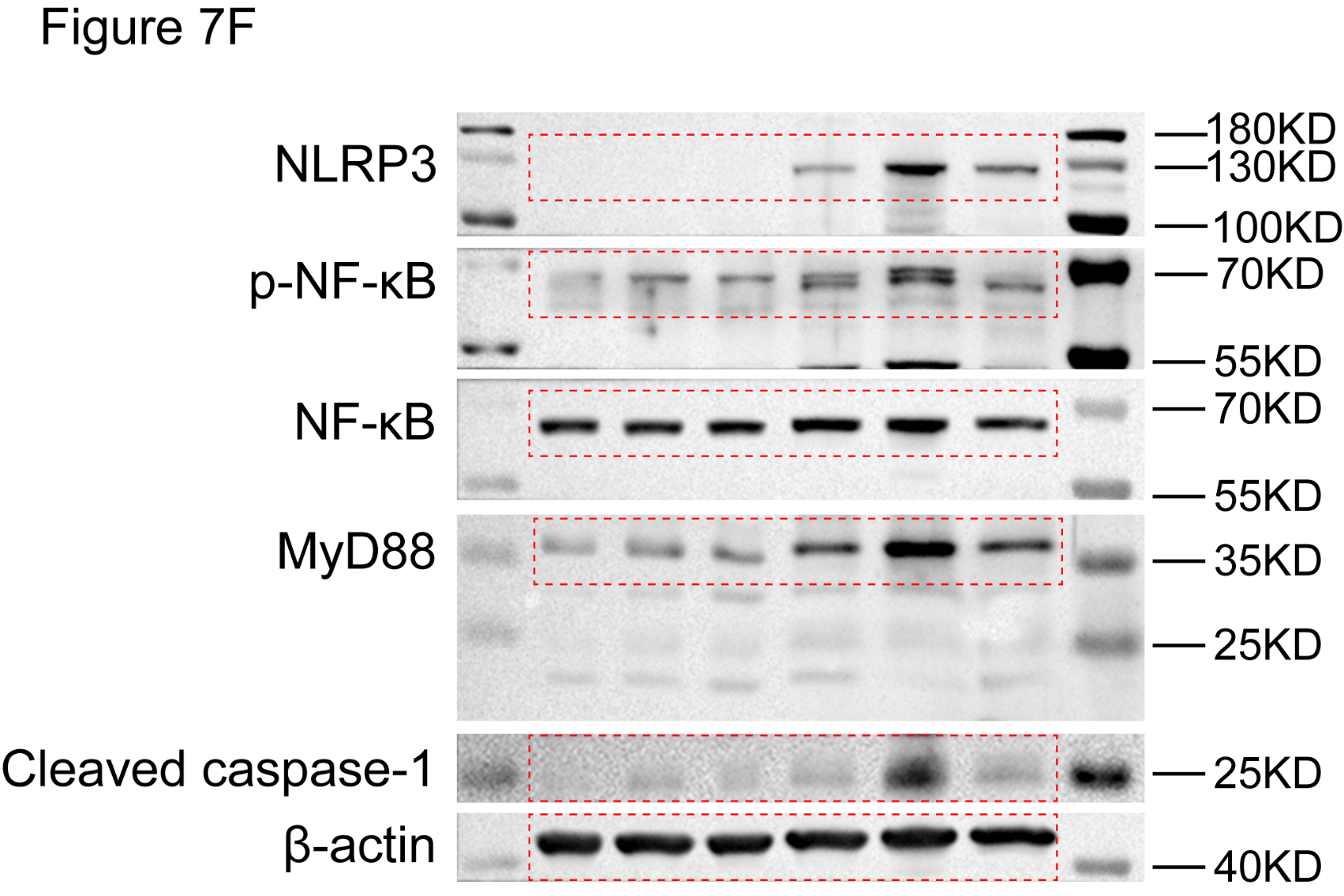


The original western blots for Figure. 7F.
